# Single-cell profiling reveals inflammatory polarization of human carotid versus femoral plaque leukocytes

**DOI:** 10.1172/jci.insight.171359

**Published:** 2023-09-08

**Authors:** Joshua Slysz, Arjun Sinha, Matthew DeBerge, Shalini Singh, Harris Avgousti, Inhyeok Lee, Kristofor Glinton, Reina Nagasaka, Prarthana Dalal, Shaina Alexandria, Ching Man Wai, Ricardo Tellez, Mariavittoria Vescovo, Ashwin Sunderraj, Xinkun Wang, Matthew Schipma, Ryan Sisk, Rishab Gulati, Jenifer Vallejo, Ryosuke Saigusa, Donald M. Lloyd-Jones, Jon Lomasney, Samuel Weinberg, Karen Ho, Klaus Ley, Chiara Giannarelli, Edward B. Thorp, Matthew J. Feinstein

**Affiliations:** 1 Division of Cardiology, Department of Medicine,; 2 Department of Pathology, and; 3 Department of Preventive Medicine at Northwestern University Feinberg School of Medicine (NUFSM), Chicago, Illinois, USA.; 4 Northwestern University Sequencing Core, Chicago, Illinois, USA.; 5 La Jolla Institute of Immunology, La Jolla, California, USA.; 6 Division of Vascular Surgery, NUFSM, Chicago, Illinois, USA.; 7 Immunology Center of Georgia, Augusta, Georgia, USA.; 8 Department of Medicine and; 9 Department of Pathology, New York University, New York, New York, USA.

**Keywords:** Cardiology, Inflammation, Atherosclerosis

## Abstract

Femoral atherosclerotic plaques are less inflammatory than carotid plaques histologically, but limited cell-level data exist regarding comparative immune landscapes and polarization at these sites. We investigated intraplaque leukocyte phenotypes and transcriptional polarization in 49 patients undergoing femoral (*n* = 23) or carotid (*n* = 26) endarterectomy using single-cell RNA-Seq (scRNA-Seq; *n* = 13), flow cytometry (*n* = 24), and IHC (*n* = 12). Comparative scRNA-Seq of CD45^+^-selected leukocytes from femoral (*n* = 9; 35,265 cells) and carotid (*n* = 4; 30,655 cells) plaque revealed distinct transcriptional profiles. Inflammatory foam cell–like macrophages and monocytes comprised higher proportions of myeloid cells in carotid plaques, whereas noninflammatory foam cell–like macrophages and LYVE1-overexpressing macrophages comprised higher proportions of myeloid cells in femoral plaque (*P* < 0.001 for all). A significant comparative excess of CCR2^+^ macrophages in carotid versus plaque was observed by flow cytometry in a separate validation cohort. B cells were more prevalent and exhibited a comparatively antiinflammatory profile in femoral plaque, whereas cytotoxic CD8^+^ T cells were more prevalent in carotid plaque. In conclusion, human femoral plaques exhibit distinct macrophage phenotypic and transcriptional profiles as well as diminished CD8^+^ T cell populations compared with human carotid plaques

## Graphical abstract

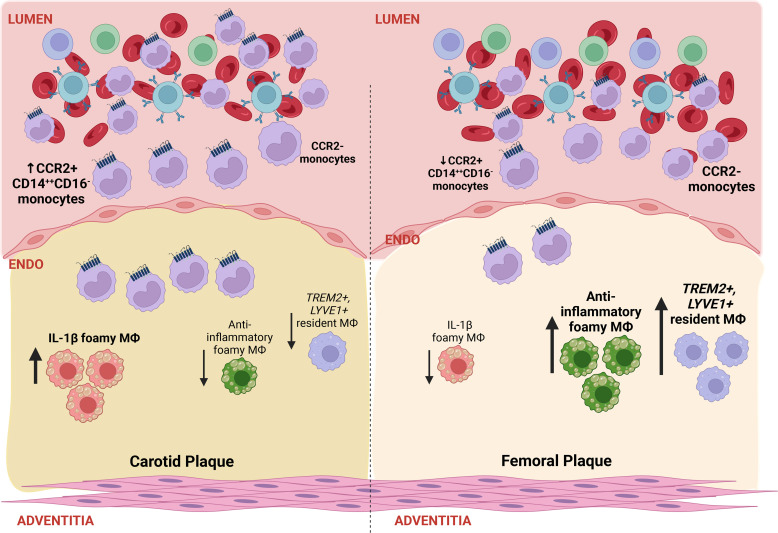

## Introduction

Vascular inflammation is a hallmark of atherosclerosis and is broadly characterized not only by the retention of cholesterol-laden lipoproteins, but also by dysregulation of innate and adaptive immune responses (1, 2). Pharmaco-interventions broadly targeting inflammation in atherosclerosis have yielded mixed results, depending on the biological and clinical target studied (3–5). These trials focused primarily on coronary and carotid-cerebral atherosclerosis-related events; clinical trial data on inflammation modulation in peripheral arterial disease (PAD) are limited and consist primarily of pleotropic lipid-lowering and antithrombotic therapy effects (6, 7). These mixed results demonstrate both the promise and challenges of broadly targeting systemic inflammation to treat and prevent atherosclerotic cardiovascular events in distinct vascular beds.

Given heterogeneity of human atherosclerosis, there is a need to move beyond singular, unified definitions of atherosclerosis (8). Carotid plaques are prone to vulnerability and rupture, leading to acute downstream effects such as acute ischemic stroke (9–11), whereas femoral atherosclerosis and resultant PAD progress gradually and generally without rupture; the majority of patients with symptomatic PAD have stable symptoms at 1 year (12–14). Recent histologic analyses provided morphologic and bulk gene expression–related corollaries to these clinical observations; carotid plaques generally have more inflammatory cells and bulk expression of genes associated with inflammatory cytokines and cell proliferation compared with femoral plaques (15–17).

The aforementioned studies have yielded key initial insights into phenotypic and functional differences between femoral and carotid arterial plaque, including distinct local immune milieus, yet several important questions remain unresolved. Whereas bulk sequencing assays provide high-level gene readouts across broad populations, single-cell analyses enable a more granular definition of cell type–specific transcriptional programs and important functional heterogeneity. Recent single-cell analyses of leukocytes in human carotid atherosclerotic plaque obtained from surgical endarterectomy revealed important and previously underappreciated features of innate and adaptive immune cells in plaques (18, 19). Meanwhile, the only prior single-cell study of human femoral plaque to our knowledge was a recent study that compared single-cell RNA-Seq (scRNA-Seq) data generated from a single femoral plaque to a publicly available carotid scRNA-Seq data set (20). Therefore, we compared the distinct, cell-specific transcriptional programs in human femoral versus carotid plaques after endarterectomy using scRNA-Seq and validated key plaque site–level differences in separate validation cohorts by flow cytometry and IHC..

## Results

### Study population.

We performed scRNA-Seq on freshly excised plaque samples from 13 distinct patients who underwent femoral (*n* = 9; 35,265 CD45^+^ cells analyzed) or carotid (*n* = 4; 30,655 cells analyzed) endarterectomy; whole plaques were digested into single-cell suspensions to optimize cell yield for sequencing. To validate key findings related to immune cell phenotypes in femoral versus carotid plaque, as well as correlates in blood, we enrolled 24 additional participants undergoing carotid (*n* = 15) or femoral (*n* = 9) endarterectomy to perform flow cytometry on plaque suspensions (prepared in the same manner as for scRNA-Seq analyses) and preoperatively obtained blood samples. We also investigated relative proportions of B and T cells in lymphoid aggregates in situ via IHC from plaque specimens of an additional 12 patients who underwent femoral (*n* = 5) or carotid (*n* = 7) endarterectomy. This yielded a total of 49 distinct patients who underwent femoral (*n* = 23) or carotid (*n* = 26) endarterectomy (Supplemental Figure 1; supplemental material available online with this article; https://doi.org/10.1172/jci.insight.171359DS1).

### scRNA-Seq of femoral and carotid plaques reveals 13 distinct leukocyte clusters.

**Figure 1:**
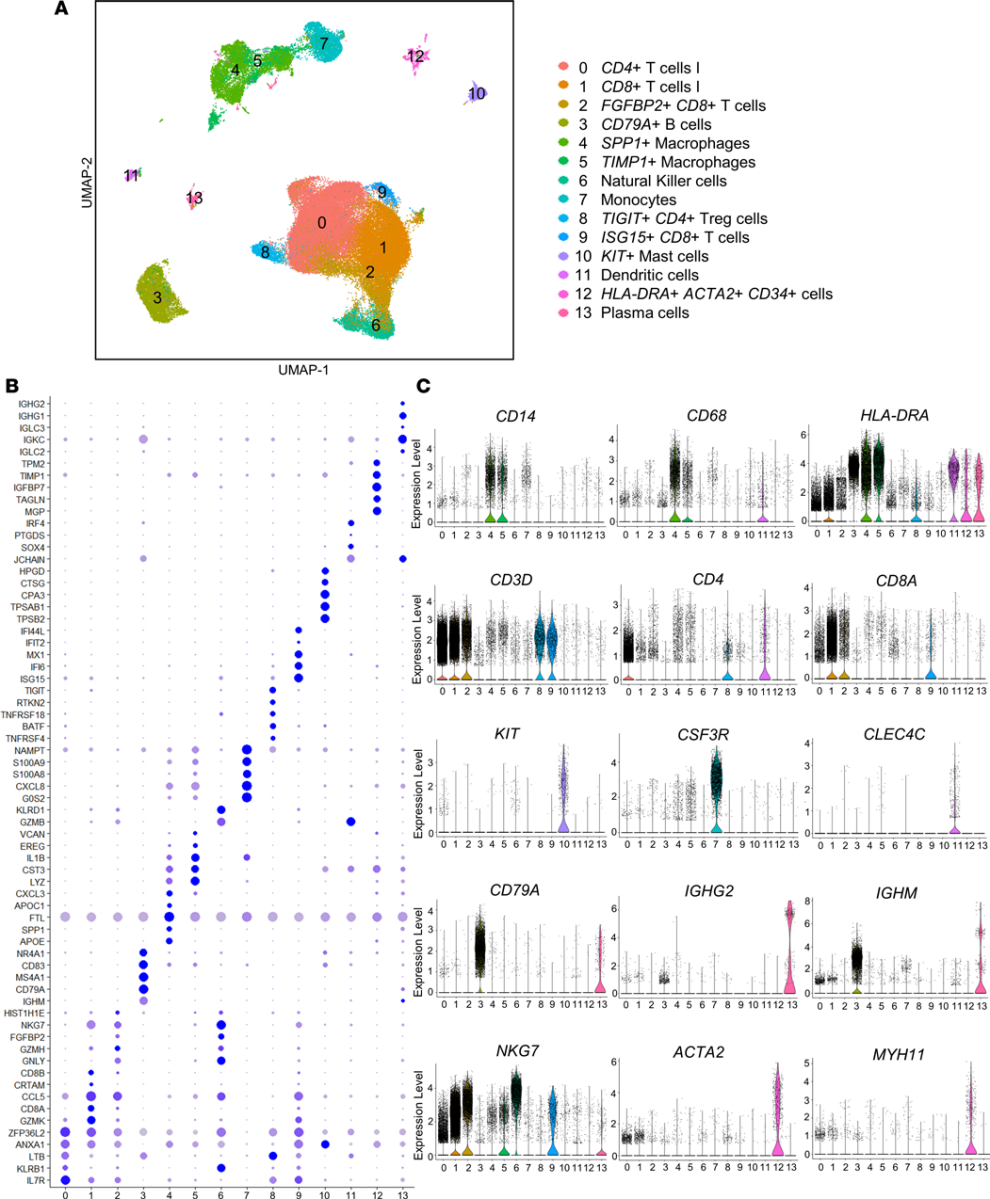
Canonical correlation analysis (CCA) clustering of CD45^+^ selected cells derived from scRNA-Seq of femoral (*n* = 9; 35,265 cells) and carotid (*n* = 4; 30,655 cells) atherosclerotic plaque reveals distinct immune cell types and populations. (**A**) CCA clustering and UMAP visualization of all femoral (*n* = 9) and carotid (*n* = 4) plaque samples for which scRNA-Seq was performed, including UMAP for overall samples colored by 14 cell types. (**B**) Dot plot of top marker genes per cluster. (**C**) Cell type identities were validated based on marker gene expression.

After quality control (Table 1 and Supplemental Figure 2), 65,920 CD45^+^ cells (30,655 carotid and 35,265 femoral) were analyzed using the Seurat package. Clustering of total CD45^+^ cells revealed 13 leukocyte cell clusters (Figure 1; clusters with sample-level and gene-specific Uniform Manifold Approximation and Projection (UMAP) overlays in Supplemental Figures 3 and 4) and 1 cluster (Cluster 12) expressing *CD34* and *ACTA2*. To validate manual cluster annotation, cell types were automatically annotated using the CellTypist package (21) to label cells against the “Immune_All_High” reference data set, which includes immune population from 20 tissues derived from 18 studies; this validated our manual cluster annotation and observed overall similar proportions of cell subsets by plaque site (Supplemental Figure 5). The 13 leukocyte clusters included 5 myeloid clusters (Clusters 4, 5, 7, 10, and 11). Clusters 4 and 5 expressed upregulated *CD14* and *CD68,* consistent with macrophages. A small mast cell population (Cluster 10) was defined by upregulated expression of *KIT, HDC, CMA1*, and *TPSAB1*, while a distinct population (Cluster 7) represented monocytes. DCs (Cluster 11) expressed *CLEC4C*.(22, 23) The 8 lymphoid clusters (Clusters 0, 1, 2, 3, 6, 8, 9, and 13) consisted of a population of B cells (Cluster 3, expressing *CD79A* and *CD22*), a smaller cluster of plasma cells (Cluster 13, expressing *IGHM, JCHAIN, IGHG*), NK cells, and 5 T cell clusters (22, 23). T cell clusters included 2 CD4^+^ T and 3 CD8^+^ T cell subsets (defined in more detail in lymphoid subclustering analyses below).

**Figure 2:**
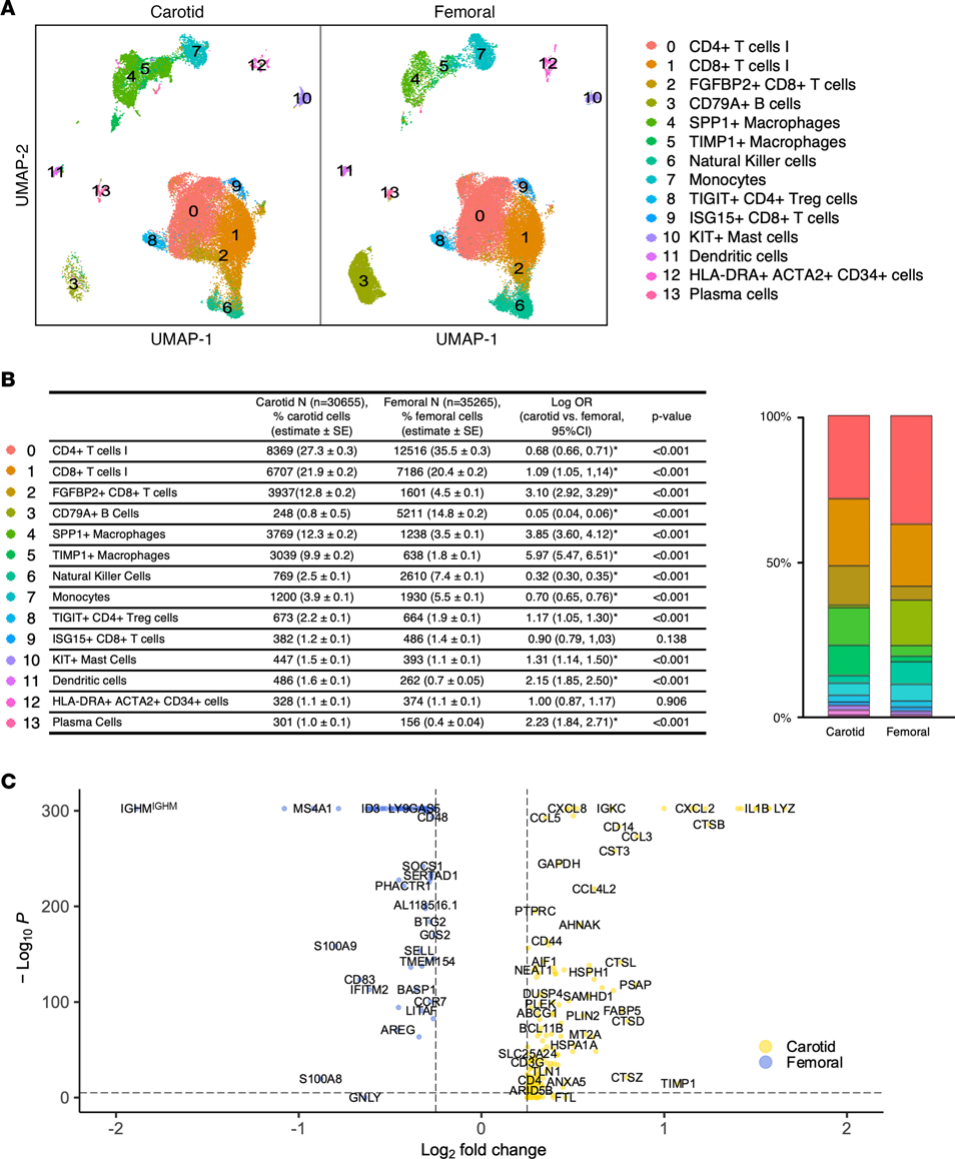
Quantitative comparison between femoral (*n* = 9; 35,265 cells) and carotid (*n* = 4; 30,655 cells) atherosclerotic plaque from CCA clustering of CD45^+^ selected cells. (**A**) UMAP visualization of separated carotid and femoral samples. (**B**) Corresponding table and the stacked bar graph of the logistic regression comparing cell proportions in carotid versus femoral plaque (log OR, 95% CI expressed). (**C**) Volcano plot of highly expressed genes for carotid and femoral plaque samples.

When comparing femoral versus carotid plaques, femoral plaques had lower proportions of macrophages and higher proportions of B cells than carotid plaque (Figure 2, A and B). Our observed proportions of CD45^+^ cells in carotid plaque that were T cells versus monocytes/macrophages were remarkably similar to those observed in 2 distinct scRNA-Seq studies of carotid atherosclerotic plaque (18, 19); no prior studies have performed such analyses in femoral plaque, precluding similar comparisons. Taken together, unique vascular bed–specific signatures between femoral and carotid plaques emerged. When investigating differential gene expression overall by plaque site for CD45^+^ cells (Figure 2C), inflammatory gene *IL1B* was one of the most comparatively overexpressed in carotid plaque, along with *CCL3*, which is a chemokine that recruits monocytes and T cells and triggers inflammatory macrophage polarization. Meanwhile, B cell–associated genes *IGHM* and *CD79A* were the most comparatively overexpressed for femoral versus carotid plaque (24). B cell–associated genes *IGHM* and *CD79A* were the most comparatively overexpressed for femoral versus carotid plaque. To further delineate heterogeneous cell clusters and cluster-specific differences in proportion and transcriptomes in femoral and carotid plaque, subsequent analyses and interpretation were based on separate myeloid- and lymphoid-specific reclustering.

### Myeloid-specific reclustering elucidates distinct macrophage profiles in femoral versus carotid plaque.

**Figure 3:**
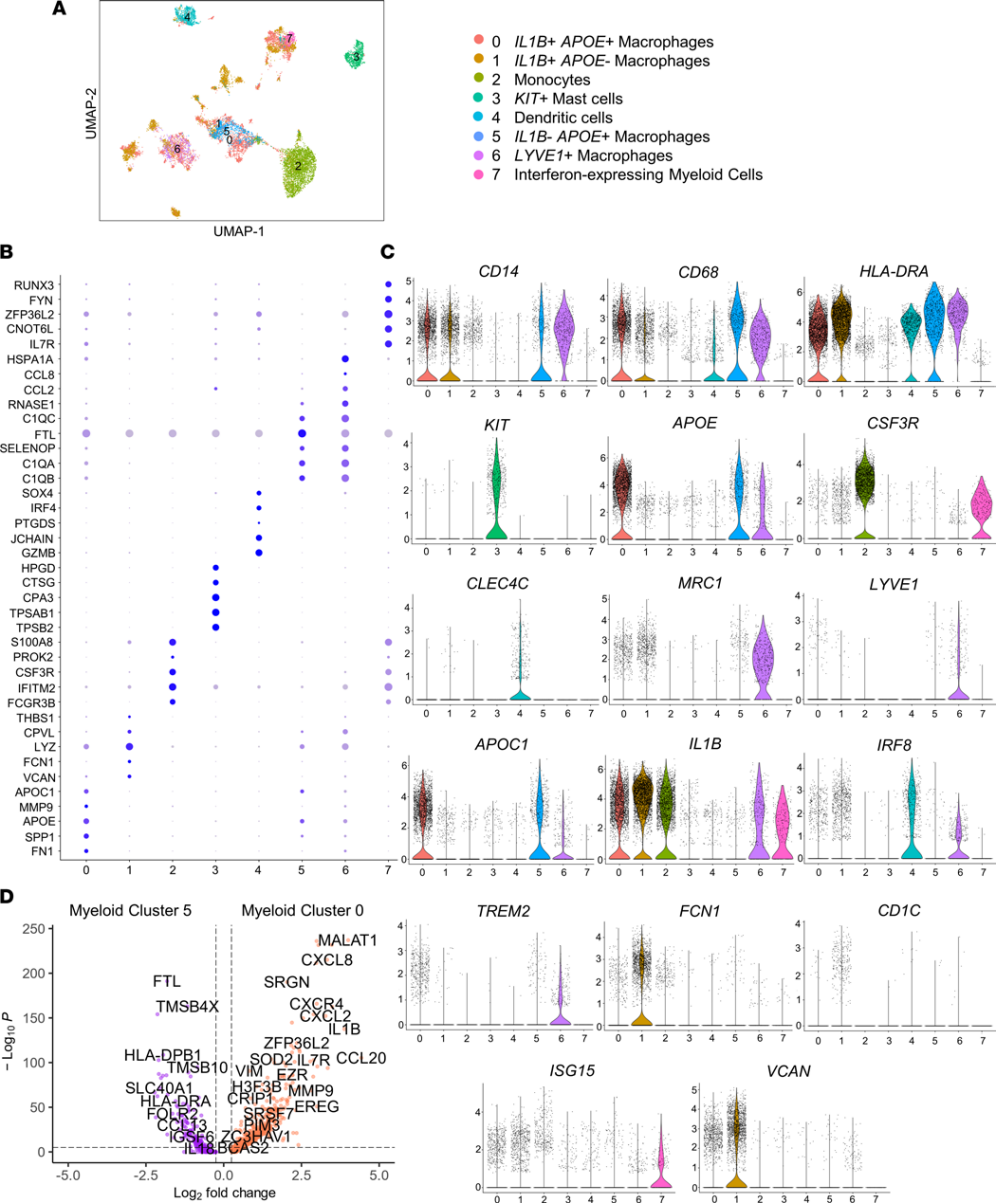
Myeloid reclustering of scRNA-Seq data reveals inflammatory foam cell–like macrophage and monocyte predominance in carotid plaque (*n* = 4; 8,941 myeloid cells) and comparative antiinflammatory and resident-like macrophage biases in femoral plaque (*n* = 9; 4,461 myeloid cells). (**A**) CCA Re-Clustering and UMAP visualization of carotid and femoral myeloid cell samples, including overlay by vascular bed revealed 8 distinct populations. (**B**) Dot plot of top marker genes per cluster. (**C** and **D**) Top marker genes per cluster are shown by violin plot, and macrophages with high *APOE* and *APOC1* expression suggestive of foam cell features consisted of 2 distinct clusters, labeled inflammatory and noninflammatory based on differential gene expression.

**Figure 4:**
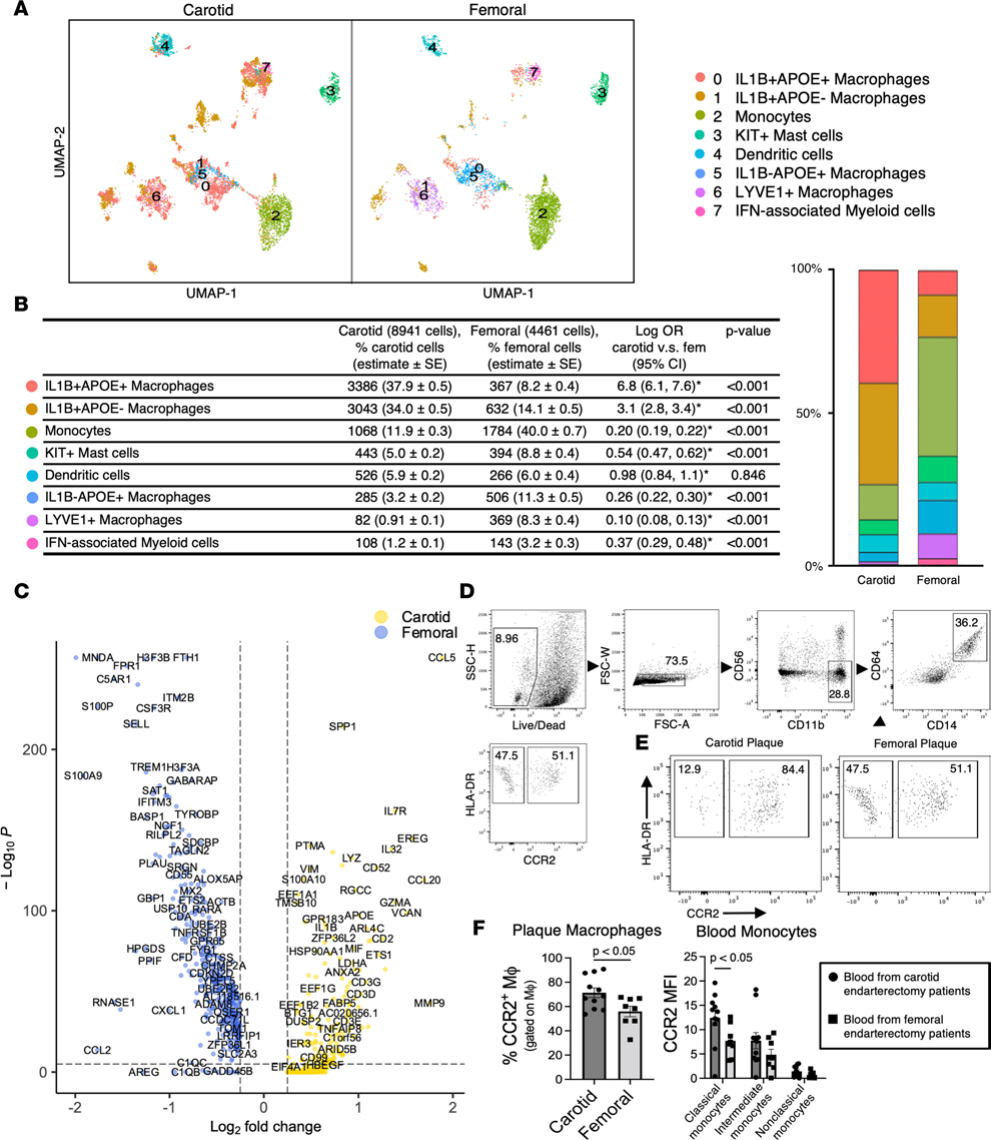
Distinct macrophage gene expression and phenotypic profiles confirmed by scRNA-Seq (8,941 carotid myeloid cells, 4,461 femoral myeloid cells) and in a validation cohort by flow cytometry. (**A**) Cell clusters as a proportion of myeloid cells were compared for carotid versus femoral samples and are displayed in UMAP visualization of separated carotid and femoral samples. (**B**) Stacked bar graph and corresponding table of the logistic regression comparing cell proportions in carotid versus femoral plaque (log OR, 95% CI expressed; *P < 0.0017, between carotid and femoral plaques that were significant at Bonferroni-corrected value. (**C**) Volcano plot of highly expressed genes for carotid versus femoral plaque overall. (**D**–**F**) Flow cytometry of digested plaque macrophages, identified as CD11b^+^CD14^+^CD64^+^HLA-DR^hi^ live cells and further distinguished based on CCR2 expression, revealed a significantly higher proportion of carotid plaque macrophages expressing CCR2 than carotid plaque macrophages (representative plot in **E**, comparison in **F**, left plot). Classical (CD14^++^CD16^–^) monocytes from blood also expressed CCR2 more highly in patients undergoing carotid versus femoral endarterectomy (**F**, right plot).

Myeloid cell subcluster analyses revealed 8 distinct populations (My.0–My.7; Figure 3 and Supplemental Figure 6). Macrophage clusters My.0 and My.5 highly expressed genes involved in lipid uptake and metabolism (*APOC1* and *APOE*), suggesting foamy macrophages (25); however, these clusters exhibited markedly distinct gene expression profiles. My.0 (*IL1B^+^APOE^+^* inflammatory foam cell–like macrophages) was enriched for inflammatory cytokine activation, with > 10-fold higher relative expression of *IL1B*, *CXCR4* (26), and *CCL20* than My.5 (*IL1B^–^APOE^+^* noninflammatory foam cell–like macrophages), which was comparatively enriched for genes involved in repair and antigen presentation. Notably, My.5 had > 4-fold higher expression of complement-related gene *C1QB* (relevant to complement’s prosurvival, efferocytosis-boosting activities in foam cells, and context-dependent activity in atherosclerotic inflammation regulation; refs. 27, 28) and > 4-fold higher expression of *RNASE1*, a regulator of vascular homeostasis that protects endothelial cells from damage-associated molecular protein effects in acute inflammation (29). Proportions of these cells comprising carotid versus femoral plaque myeloid cells differed significantly (Figure 4, A and B). Inflammatory foam cell–like macrophages (My.0) comprised a significantly higher proportion of overall myeloid cells in the carotid versus femoral plaque (37.9% versus 8.2%; log odds ratio [OR] 6.8 [6.1–7.6], *P* < 0.001], whereas noninflammatory foam cell–like macrophages (My.5) were far less common in carotid versus femoral plaque (3.2% versus 11.3%; log OR 0.26 [0.22–0.30], *P* < 0.001).

We also identified a separate population of *LYVE1^+^* macrophages (My.6; Figure 3) also expressing high levels of *TREM2*, a gene previously implicated in antiinflammatory macrophages in murine atherosclerosis (30–32). This *LYVE1^+^* macrophage cluster comprised a roughly 10-fold–higher proportion of the myeloid cells in femoral versus carotid plaque (8.3% versus 0.9%, log OR 10.1 [7.7–12.5], *P* < 0.001); this represented 26.7% of femoral plaque macrophages versus 2.1% of carotid plaque macrophages (*P* < 0.001), suggesting a greater inflammatory potential of macrophages enriched in carotid plaques (Figure 4, A–C). Carotid plaques were also comparatively enriched for macrophages with high *IL1B* but low *APOE* or *APOC1* expression (My.1; *IL1B^+^APOE^–^* inflammatory non–foam cell–like macrophages; 34.0% of total myeloid cells in carotid plaque versus 14.1% in femoral plaque; *P* < 0.001).

**Figure 5:**
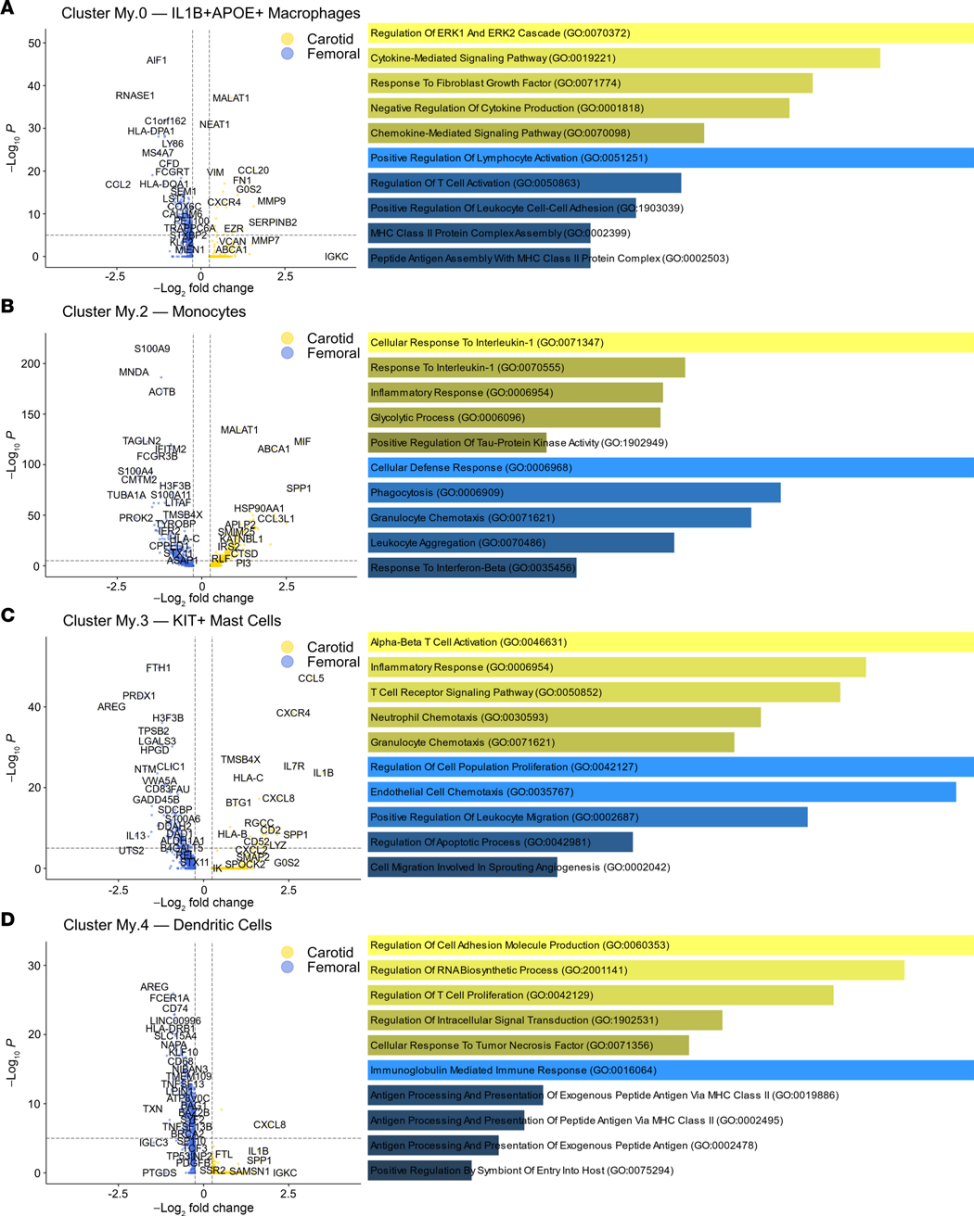
Differential gene expression analyses of femoral versus carotid myeloid clusters reveal comparative homeostatic, inflammation-regulating biases in femoral plaque. (**A**–**D**) Volcano plots of differential gene expression and gene ontology (GO) analyses of biological processes in femoral versus carotid plaque for inflammatory foam cell–like macrophages (**A**), monocytes (**B**), mast cells (**C**), and DCs (**D**) depict differential gene expression for carotid and femoral plaques.

The enrichment of fewer inflammatory macrophage phenotypes in femoral versus carotid plaque and the inflammatory nature of foam cell–like macrophages largely corresponded to distinct gene expression profiles (Figure 5). The My.0 cluster in femoral plaques had significantly higher expression of genes involved in cell-mediated immune response and monocyte recruitment (*HLA-DPA1, HLA-DQA1, CCL2*), as well as homeostatic (*RNASE1*) and complement-related (*C1QA*) genes, reflected in antigen processing and presentation enrichment identified by gene ontology (GO). In contrast, the carotid My.0 cluster significantly exhibited a potent inflammatory profile, with GO analyses demonstrating enrichment of inflammatory cytokine and chemokine responses as well as overexpression of individual genes — including *MMP9*, which is associated with coronary and carotid plaque instability and plaque neo-angiogenesis (33–38), and *CCL20*, a potent chemokine-encoding gene previously named macrophage inflammatory protein 3α, which is highly induced by inflammatory stimuli and exerts context-dependent inflammatory effects (39).

A separate myeloid population (My.2; Figure 3) minimally expressing *CD14* and *CD68* (unlike My.0, My.1, My.5, and My.6), nor *APOC1* and *APOE* (unlike foam cell–like macrophage clusters My.0 and My.5) was marked by high *CSF3R*, *S100A8*, and *S100A9* expression and exhibited a gene expression profile most consistent with monocytes (21). In this cluster, 2 of the most overexpressed genes in carotid versus femoral plaque (both > 6-fold higher expression) were *MIF*, a potent acute inflammatory signaling molecule implicated in endotoxinemia and sepsis (40), and *SPP1* (osteopontin), a broadly activating cytokine with roles in acute and chronic inflammation (41, 42). In contrast, genes involved in inflammation-regulating, proapoptotic functions were comparatively upregulated in femoral plaque My.2 (Figure 5B); the 3 most comparatively overexpressed genes (*MNDA, S100A8*, and *S100A9*) were inflammation-response genes with context-dependent regulatory functions. *MNDA* is an IFN-induced gene with regulatory effects including regulation of myeloid differentiation (43) and induction of apoptosis (44, 45) as a means of decreasing acute inflammation in conditions such as sepsis (43, 46). *S100A8/9* are likewise stress-induced polyfunctional genes with context-dependent functions, including regulation of inflammation, apoptosis, and tissue repair (47–49).

To determine whether the general differences in monocyte/macrophage and T cell transcriptional phenotypes corresponded to changes in cell types based on canonical protein markers, we prospectively enrolled an additional 24 patients undergoing femoral (*n* = 9) or carotid (*n* = 15) endarterectomy for flow cytometry analysis of plaque and paired blood (Table 2). Patient clinical and demographic characteristics were similar between those undergoing femoral versus carotid endarterectomy — including similar age and sex distribution as well as prevalence of diabetes, hypertension, and statin use between femoral and carotid endarterectomy groups. The exception was that current smoking was more common in patients with femoral endarterectomy, consistent with the higher prevalence of smoking among patients with PAD versus CAD or stroke (50, 51). Plaque specimens were digested into single-cell suspensions using the same digestion methods as for scRNA-Seq analyses, gated on macrophages (CD11b^+^CD14^+^CD64^+^HLA-DR^hi^ cells), and further subclassified based on expression of CCR2 (Figure 4, D–F), a chemokine receptor expressed on monocyte-derived inflammatory macrophages (52–54). Consistent with our overall comparisons of myeloid phenotypes in the scRNA-Seq data (with carotid plaques demonstrating a inflammatory myeloid signature), we observed a significantly higher proportion of CCR2^+^ macrophages in carotid (71.4% of total plaque macrophages) versus femoral (56.9%) plaque (*P* = 0.03). Circulating classical (CD14^++^CD16^–^) monocytes from patients with carotid plaques also expressed high levels of CCR2 (mean fluorescence intensity [MFI] = 12.8 ± 1.4 relative fluorescence units [RFU]) versus blood classical monocytes from patients undergoing femoral endarterectomy (MFI = 7.0 ± 1.0 RFU) (*P* = 0.008).

### Mast cells and DCs exhibit inflammatory transcriptional bias in carotid versus femoral plaque.

Myeloid clusters My.3 and My.4 were defined *KIT*^+^ mast cells and DCs (marked by *CLEC4C* and *IRF8* expression), respectively (18, 55, 56). Mast cells comprised a lower proportion of myeloid cells in femoral plaque, whereas DC proportions were similar in carotid and femoral plaques (Figure 4, A and B). However, we observed striking transcriptional differences for these cells in femoral versus carotid plaque. Mast cells exhibited a comparatively potent inflammatory transcriptional signature in carotid versus femoral plaque, with > 6-fold higher expression of *IL1B* and *CCL5* in carotid versus femoral mast cells (Figure 5C). Amphiregulin (*AREG*), a master regulator of tissue homeostasis and repair (57), was the most highly overexpressed gene in femoral versus carotid mast cells and DCs (Figure 5D). Femoral DCs also expressed high levels of *HLA-DRB1* and *CD74* compared with carotid DCs, suggestive of an enhanced antigen-presenting signature in femoral plaque.

### Lymphoid reclustering suggests inflammatory and cytotoxic T cell bias in carotid plaque versus B cell bias in femoral plaque.

**Figure 6:**
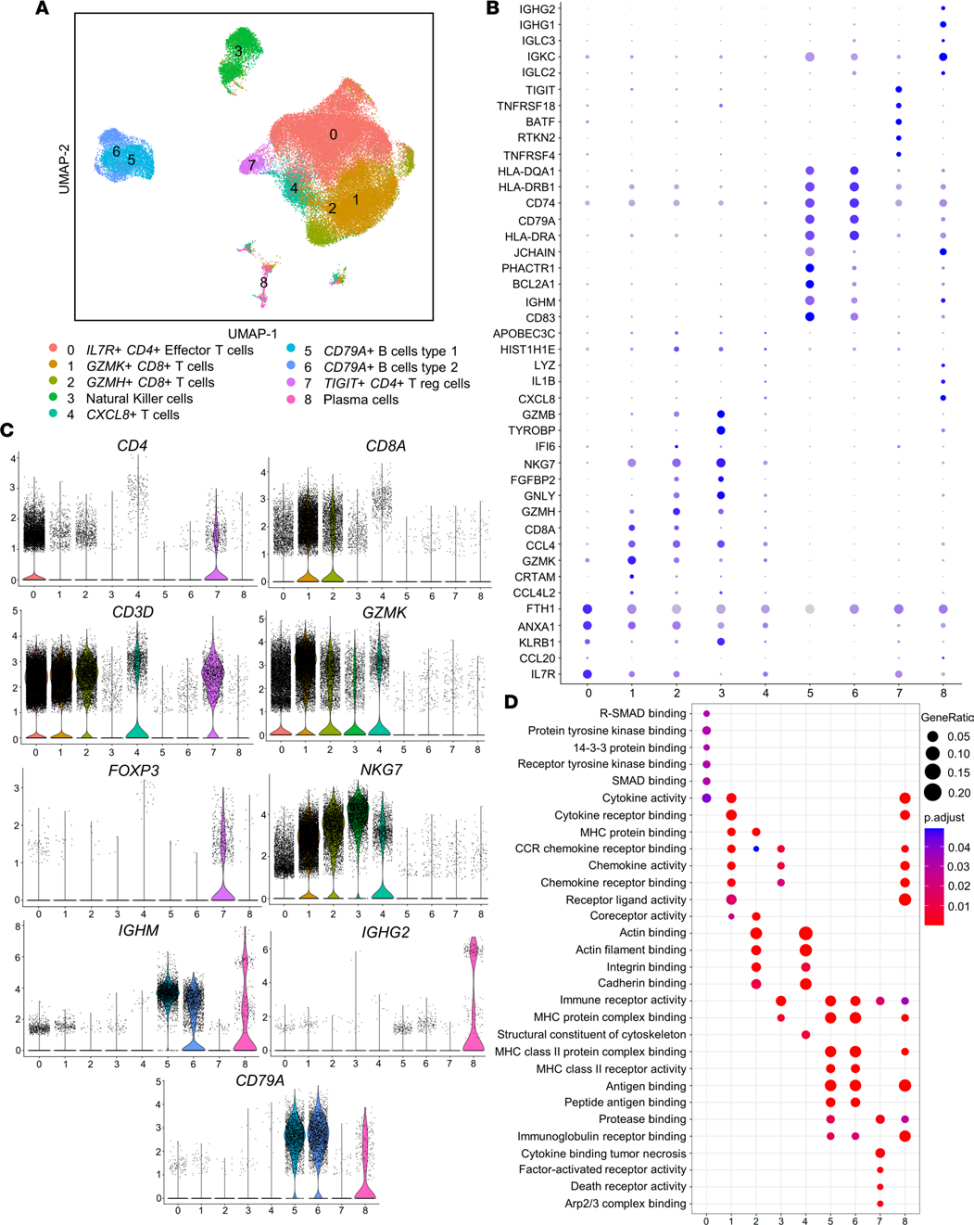
Lymphoid reclustering of scRNA-Seq data reveals T cell predominance and cytotoxicity-associated gene expression in highly prevalent T cell clusters. (**A**) Visualization of carotid and femoral lymphoid cells revealed 9 distinct populations. (**B**) Highly expressed genes are visualized with the dot plot. (**C**) Top marker genes per cluster are shown by violin plot. (**D**) Related gene set enrichment shown by the dot plot.

Lymphoid cell–specific reclustering revealed 9 distinct lymphoid populations (Ly.0–Ly.8; Figure 6). Clusters were determined using a combination of previously defined cluster-specific genes, reference data sets of combined human protein and transcript single cell transcriptomes (21, 58), and gene set enrichment analyses (GSEA). Clusters Ly.0–Ly.2, Ly.4, and Ly.7 all highly expressed *CD3D* and represent T cell clusters, with Ly.0 and Ly.7 expressing *CD4*, representing CD4^+^ T cells (and Ly.7 expressing *TIGIT* and *FOXP3* as a Treg cluster). Ly.1 and Ly.2 highly expressed *CD8* as CD8^+^ T cells, most highly expressing cytotoxicity markers *GZMK* and *GZMH*, respectively. GSEA suggested high composition of T helper type 1 (Th1) cells in Ly.0, which also had high *IL7R* expression (previously observed in Th1 effector cells; ref. 59). Conversely, Ly.7 highly expressed *FOXP3* and other Treg-related genes without inflammatory or cytotoxicity-associated gene expression profiles. Cluster 4 represented a group of *CD3*-expressing T cells that did not have clear *CD4* or *CD8* expression but had comparatively high expression of *CXCL8* (60, 61). Cluster 3 highly expressed *NKG7* and did not express *CD3D* (while also having minimal *CD4* and *CD8* expression), thus representing NK cells. Clusters 5 and 6 were *CD79*-expressing B cells, with distinctions in gene expression between these 2 B cell clusters suggesting that Ly.5 is type 1 B cells (B1) and Ly.6 is type 2 B cells (B2). B1 cells have been implicated in the secretion of atheroprotective IgM (62), whereas B2 cells follow more conventional paradigms driven by interaction with T cells in germinal centers to form high-affinity antibodies orchestrating humoral responses (63). Ly.5 overexpressed *BHLHE41* and *ZBTB32* — 2 transcriptome markers of B1 cells, and Ly.6 overexpressed Fcer2, which has recently been established as a B2 gene marker (64).

**Figure 7:**
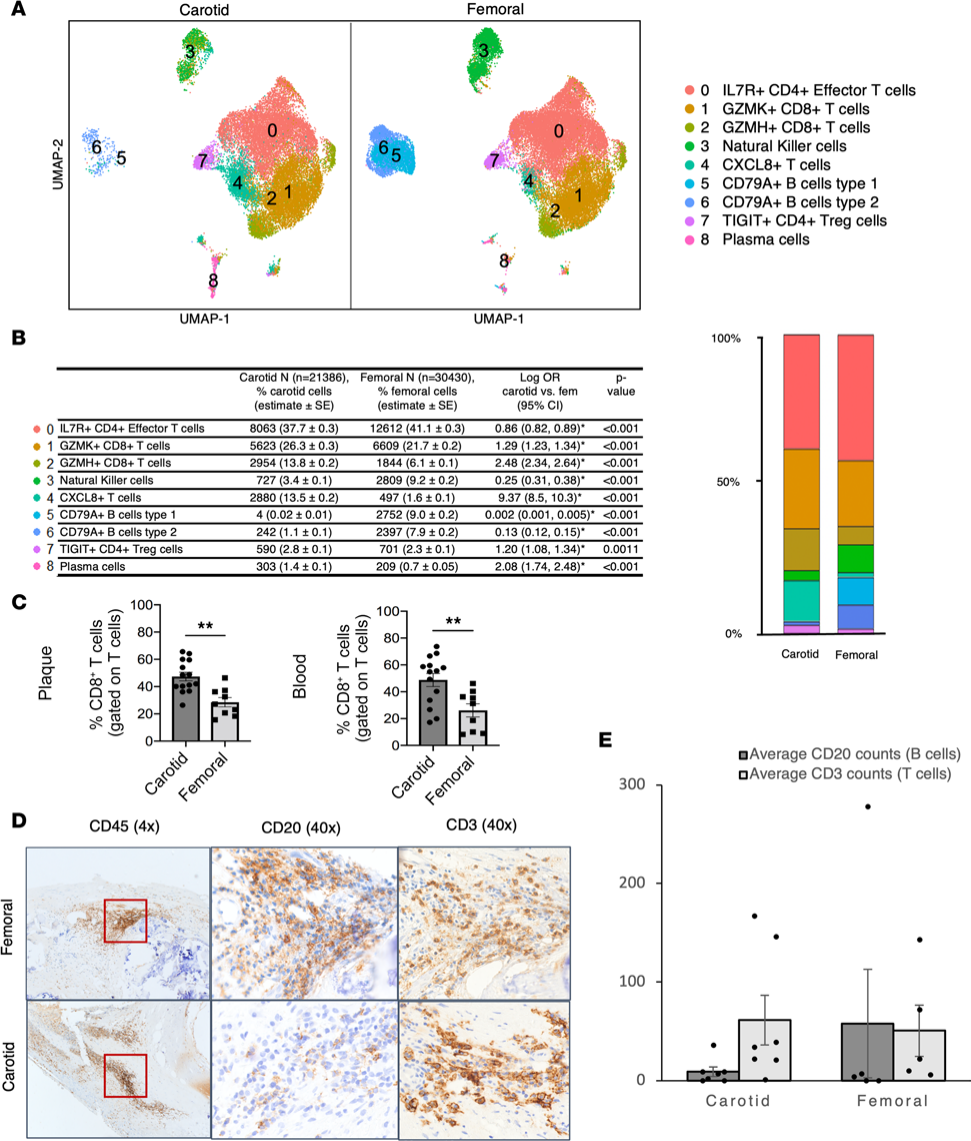
Carotid plaque exhibits comparative cytotoxic CD8^+^ T cell bias, whereas B cells are more prevalent in femoral plaque. (**A** and **B**) UMAP visualization of separated carotid and femoral samples with stacked bar graph and corresponding table of the logistic regression comparing cell proportions in carotid versus femoral plaque (log OR, 95% CI expressed). *P < 0.0017, differences between carotid and femoral plaques that were significant at Bonferroni-corrected value. (**C**) Flow cytometry of T cells from paired plaque and blood samples revealed comparative excess in CD8^+^ T cells (as a proportion of overall T cells) in plaque and blood from carotid endarterectomy patients (**P < 0.05 using 2-tailed *t* test). (**D** and **E**) In situ determination and numbers of B and T cells per high-powered field in intraplaque leukocyte aggregates. Magnification, 4× (left) and 40× (middle and right).

The vast majority of lymphoid cells in both femoral and carotid plaque were T cells (Figure 7, A and B), with approximately equal proportions of CD4^+^ (Ly.0 + Ly.7) and CD8^+^ T cells (Ly.1 + Ly.2) in carotid plaque, as observed previously in a separate cohort (19). When comparing cell proportions in femoral versus carotid plaque, the most notable difference in T cell subsets was a comparatively higher proportion of cytotoxic CD8^+^ T cells in carotid versus femoral plaque (Ly.1 + Ly.2: 40.1% versus 27.8%; *P* < 0.001). The CD8^+^ T cell enrichment in carotid plaque was validated by flow cytometry in the prospective cohort of patients undergoing carotid (*n* = 15) or femoral (*n* = 9) endarterectomy (Figure 7C and Supplemental Figure 8 for T cell gating); interestingly, the higher CD8^+^ T cell proportion was also observed in the blood of patients with carotid plaques. Meanwhile, femoral plaques were highly enriched in B cells compared with carotid plaque (9.0% versus 0.02% for Ly.5 and 7.9% versus 1.1% for Ly.6; *P* < 0.001). To explore lymphoid cell clustering in situ, we quantified T cell and B cell aggregates in 12 separate patients who underwent carotid (*n* = 7) or femoral (*n* = 5) endarterectomy and observed that T cells were the most populous cell type in these aggregates in femoral and carotid plaques, although 1 femoral plaque had a marked B cell aggregate (278 CD20^+^ B cells and 143 CD3^+^ T cells per high-powered field; Figure 7, D and E).

**Figure 8:**
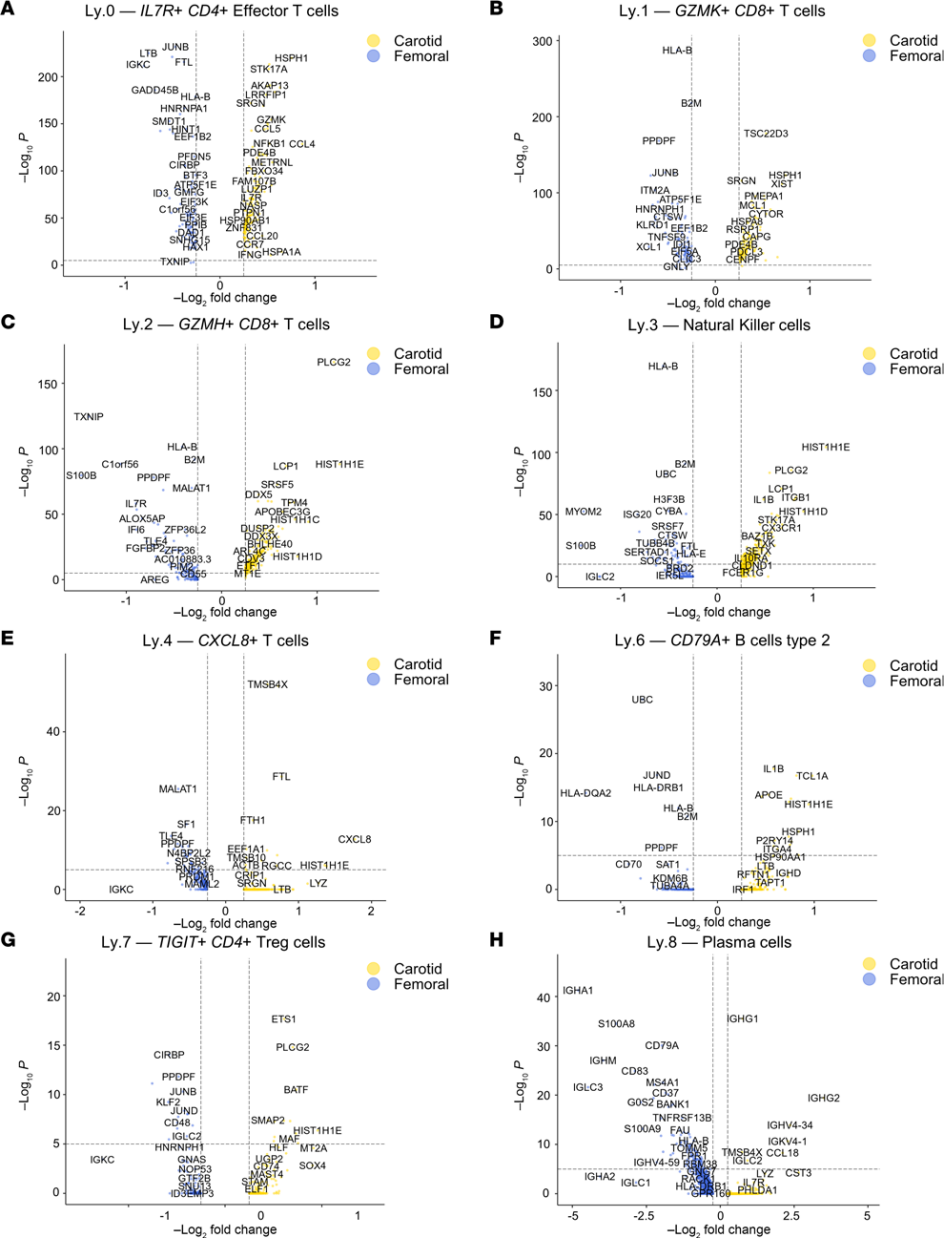
Differential gene expression analyses of femoral versus carotid lymphoid clusters demonstrate comparative inflammation-regulating bias in femoral plaque. (**A**–**H**) Volcano plots of differential gene expression in femoral versus carotid plaque for *IL7R*^+^ CD4^+^ Effector T cells (**A**), GZMK^+^CD8^+^ T cells (**B**), GZMH^+^CD8^+^ T cells (**C**), NK cells (**D**), CXCL8^+^ T cells (**E**), CD79A^+^ B cells type 2 (**F**), TIGIT^+^ CD4^+^ Tregs (**G**), and plasma cells (**H**). CD79A^+^ B cells type 1 were not included due to the insufficient cell number in carotid plaques.

Differential gene expression analysis suggested an inflammatory bias in several carotid plaque clusters (Figure 8). All 4 of the largest lymphoid clusters (Ly.0–Ly.3, representing a heterogeneous cluster of *IL7R*^+^ CD4^+^ T cells [Ly.0], 2 cytotoxic CD8^+^ T cell clusters [Ly.1 and Ly.2], and NK cells [Ly.3]) overexpressed the antigen presentation–associated gene *HLA-B* in femoral plaque. Ly.2, 1 of 2 CD8^+^ T cell clusters (2-fold higher in carotid plaques), overexpressed several stress-sensing genes with regulatory functions. These included *TXNIP*, a metabolic stress sensor with context-dependent actions including inhibition of cell proliferation (65) and suppression of inflammation (66), and *S100B* (discussed above). The potentially novel longevity-associated gene *c1orf56* (67) was also overexpressed in Ly.2 femoral versus carotid plaque. In Ly.4 (*NKG7*^+^ T cells), *CXCL8* — a potent and broadly inflammatory cytokine-encoding gene (68) — was upregulated in carotid plaque. B2 cells had a more inflammatory signature in carotid plaque compared with femoral plaque, with significant overexpression (in carotid plaque) of *IL1β* and *SPP1*, the latter encoding for a potent inflammatory cytokine (41) also overexpressed in carotid myeloid cluster 2.

## Discussion

In this study, we observed important differences in the immune cell landscape and cell-specific divergence in gene expression and phenotype for femoral versus carotid atheroma obtained at endarterectomy. Our findings provide insights into cellular composition and heterogeneity of femoral and carotid plaques, informing on transcriptional reprogramming that may underlie histopathologic (15–17) and clinical (12–14) stability of femoral versus carotid plaque (9–11).

Femoral plaques largely exhibited homeostatic gene expression pattern signatures compared with more broadly inflammatory signatures of carotid plaques, a distinction that was especially apparent in myeloid clusters. Polarization of macrophages highly expressing *APOC1* and *APOE*, suggestive of foamy macrophages, was starkly different by site. Inflammatory *IL1B*^+^ foam cell–like macrophages comprised over one-third of carotid myeloid cells, outnumbering carotid noninflammatory (*IL1B*^–^) foam cell–like macrophages more than 10-fold. Conversely, noninflammatory foam cell–like macrophages outnumbered inflammatory foam cell–like macrophages in femoral plaques. These findings were mirrored in proportions of other monocyte and macrophage subtypes. *LYVE1*^+^ macrophages, which play important roles in maintaining homeostasis — including roles via regulation and resolution of inflammation — were approximately 10-fold higher in femoral plaques (69). We confirmed macrophage plaque site–divergent changes in cell frequency and phenotype via flow cytometry in a separate validation cohort of 18 patients undergoing endarterectomy, observing that CCR2^+^ macrophages, generally reflecting activated monocyte origin, comprised a significantly higher proportion of macrophages in carotid versus femoral plaque. Interestingly, we also observed significantly higher CCR2 expression in blood CD14^++^CD16^–^ monocytes of patients undergoing carotid endarterectomy. Taken together, our observations in carotid plaque of comparative monocyte excess, macrophage inflammatory bias, and divergent plaque and blood CCR2 expression on macrophages and monocytes raise the possibility of distinct monocyte/macrophage polarization profiles by plaque site.

We likewise observed a inflammatory T cell bias in carotid versus femoral plaques. Our observed proportions of CD4^+^ and CD8^+^ T cells in carotid plaque mirrored those observed previously in a separate cohort (19), underscoring reproducibility. Our sequencing and flow data in femoral plaque add substantially to this existing carotid plaque–focused literature on intraplaque T cell phenotypes, since we observed consistently lower proportions of CD8^+^ T cells — which highly expressed cytotoxicity markers — in femoral versus carotid plaque. Furthermore, similar to our macrophage findings, we observed comparative overexpression in femoral plaques of inflammation-regulating genes (including *TXNIP*) versus carotid plaque overexpression of inflammatory genes such as *CXCL8*.

Our B cell–related findings were unanticipated. Although T cells have been reported to be the predominant lymphoid cell population in coronary, aortic, and carotid plaques (19, 70, 71), there is evidence from experimental models and human data that B cells play complex roles in atherosclerosis, with 2 overarching B cell subsets that have divergent functions (70, 72, 73). These subtype-specific roles of B cells may explain inconsistent experimental and clinical findings related to effects of broader B cell modulation on atherosclerosis (74–76); net effects of nonspecific B cell–targeted interventions may depend on the extent to which (putatively atheroprotective) B1 versus (atherogenic) B2 cells are preferentially targeted (70). In this context, our findings related to specific B cell subsets in carotid versus femoral plaque are interesting. There were > 60× more B2 cells compared with B1 cells in carotid plaque, whereas there were actually fewer B2 cells than B1 cells in femoral plaque. Our findings raise the possibility that net effects of B cells in different plaque microenvironments may vary considerably; carotid plaques have an atherogenic, inflammatory B2 bias and femoral plaques have a more balanced B1 and B2 cell presence, with a resulting stronger signature of regulatory B1-type functions. These observations, if replicated in future studies of diverse human plaque microenvironments, would have important implications for atherosclerosis location–specific interventions targeting broad and specific B cell niches.

Taken together, our findings suggest a comparative homeostatic noninflammatory bias in femoral versus carotid plaque that exists across several leukocyte phenotypes. An important resulting question is why these stark differences may exist in femoral versus carotid plaque sites, despite both being large arteries — and how these distinctions may inform more site-specific therapeutic approaches in addition to currently indicated lipid lowering. Several possibilities exist and elicit hypotheses warranting future study. One possibility is that distinct metabolic microenvironments contribute to epigenetic programs favoring upregulation of prohomeostatic genes in femoral plaques (77–79), versus a proinflammatory bias in inflammatory macrophages in carotid plaque. This question warrants further study in future analyses of plaque site–specific functional metabolism. Other possibilities include differences in site-specific mechanical and shear forces (80–85) priming the immune microenvironments in different ways, with potential mechanoimmunologic effects on myeloid and lymphoid cell activation and hematopoietic niche reconstruction (86–89). Differential femoral versus carotid plaque endothelial permeability to immune cell migration from the vascular lumen into plaque is another possible explanation for our findings, which revealed relative predominance of monocytes and inflammatory foam cell–like macrophages in carotid plaques versus a comparative antiinflammatory and resident macrophage signature in femoral plaques. These hypotheses highlight the need for models that trace cell lineage, pair simultaneously collected blood samples with plaque from these diverse vascular beds, and recapitulate plaque site differences in migration and margination of immune cells in plaque. Such studies could also investigate mechanisms of immune priming and cell-to-cell interactions, detailed investigation into inflammation-resolving biology in unique atheroma microenvironments, and chemoattraction of cells to diverse plaque sites.

### Limitations.

Our study has important limitations. To investigate plaque from live humans, we analyzed endarterectomy samples from people undergoing femoral or carotid endarterectomy for clinical indications, with potential sources of confounding results. Surgical indications differ widely for femoral versus carotid endarterectomy given distinct underlying physiologies and clinical sequelae, and differences in comorbidities between femoral and carotid endarterectomy groups may further contribute to confounding. Although our femoral and carotid endarterectomy groups were similar in age, sex, statin use, and presence of hypertension or diabetes, smoking was far more common among patients undergoing femoral endarterectomy. While this reflects a potential confounder (e.g., if smoking contributes to the distinct immune profiles of femoral versus carotid plaque), it also reflects the clinical reality of smoking being more prevalent among patients with PAD than those with other atherothrombotic sequelae such as CAD or stroke (50, 51). Heterogeneity within groups also may have affected the results; for instance, among patients undergoing femoral endarterectomy, surgical indications varied from chronic claudication to ulcer or gangrene, and presence of occlusive disease at or near the endarterectomy site likewise varied among patients. While these differences in surgical indication and clinical characteristics such as smoking may reflect common precipitants and sequelae of femoral versus carotid plaque biology, they are nevertheless important potential sources of confounding, as are the lack of data regarding potentially relevant variables such as circulating inflammatory markers that may differ between groups. A separate concern relates to potential batch effects from samples being harvested and undergoing sequencing reactions at different times, with potential differential effects on early response genes. Although we aimed to correct for these with Harmony, a software package used for batch-effect correction of scRNA-Seq data (90), and observed overall good sample- and plaque site–level integration, residual confounding related to processing and batch effects remains possible.

As with many single-cell analyses of relatively rare plaque specimens obtained from humans in vivo (18, 19, 91), our sample size was limited, with the potential to adversely affect generalizability of our plaque site–specific conclusions. We profiled 35,265 femoral plaque CD45^+^ cells derived from 9 patient specimens and 30,655 carotid CD45^+^ cells derived from 4 patient specimens. However, our number of individual cells analyzed is larger than numbers from recent single-cell analyses of carotid plaque (scRNA-Seq on 3,282 and 7,169 cells; refs. 18, 19) and the only prior scRNA-Seq study of femoral plaque, to our knowledge, which compared scRNA-Seq data from a single femoral plaque specimen with data from an existing carotid plaque scRNA-Seq data set (20). The potential generalizability of our findings is further supported by (a) confirmatory flow cytometry and IHC in distinct validation cohorts and (b) similarity of our observed T cell and myeloid cell proportions in carotid plaques to those observed in prior scRNA-Seq studies (18, 19). Nevertheless, an important limitation is low *CCR2* expression throughout our scRNA-Seq data set (although 5-fold higher among carotid than femoral myeloid cells), meaning that our flow-based validation using CCR2 reflects a broad validation of inflammatory, activated monocyte and macrophage profiles in carotid versus femoral plaque. Additional markers such as LYVE-1 would have been useful to further validate macrophage populations and should be used in future studies. Still, our plaque site–specific differences are consistent with recent histologic studies, which observed comparatively more inflammatory cells and higher bulk expression of inflammation-potentiating genes in carotid plaque compared with femoral plaque (15–17). A separate, major limitation is that, while our investigation surveys specific immune phenotypes and transcriptomes in femoral versus carotid plaque, causal experiments and functional assays will be critical to validate and extend these findings in future studies.

### Conclusion.

In conclusion, we observed several plaque site–specific single-cell immune cell gene expression profiles in human femoral versus carotid plaque. These included comparatively increased inflammatory macrophage activity and CD8^+^ T cell bias in carotid versus femoral plaque. Other notable findings included a comparative B cell bias and overexpression of inflammation-regulating genes in several leukocyte clusters in femoral versus carotid plaque. These findings inform on the immunology underlying distinct clinical courses of femoral versus carotid atherosclerosis and suggest targets for future experimental models of atherosclerotic inflammation resolution.

## Methods

### Overview of study design and participants.

All analyses involved freshly excised plaque samples obtained intraoperatively from patients undergoing clinically indicated femoral or carotid endarterectomy at Northwestern Medicine (Chicago, Illinois, USA) (total *n* = 49; Supplemental Figure 1). These samples were obtained in accordance with Northwestern University IRB–approved studies 205451 (cohort 1 of patients who had femoral and carotid endarterectomy: collection of deidentified fresh endarterectomy specimens for scRNA-Seq) and 211811 (cohort 2 of patients who had femoral and carotid endarterectomy: preoperative collection of blood and collection of fresh endarterectomy specimens for flow cytometry and histology, with detailed clinical data). Our primary analyses compared scRNA-Seq–derived transcriptional programs of leukocytes in femoral versus carotid plaque from different patients undergoing femoral or carotid endarterectomy. Plaques were also analyzed by flow cytometry, and in situ IHC was performed on plaque obtained from different patients undergoing femoral or carotid endarterectomy. The most common surgical indication for carotid endarterectomy was severe carotid stenosis without stroke, and the most common surgical indication for femoral endarterectomy was claudication (Tables 1 and 2). The first protocol involved scRNA-Seq of freshly excised, deidentified plaque samples from 13 distinct patients who underwent femoral (*n* = 9; 35,265 CD45^+^ cells analyzed) or carotid (*n* = 4; 30,655 cells analyzed) endarterectomy. To validate key findings related to immune cell phenotypes in femoral versus carotid plaque, as well as correlates in blood from the same patients, we then prospectively enrolled 24 additional patients undergoing carotid (*n* = 15) or femoral (*n* = 9) endarterectomy to perform flow cytometry on plaque suspensions (prepared in the same manner as for scRNA-Seq analyses) and preoperatively obtained blood samples. We also investigated relative proportions of B and T cells in lymphoid aggregates in situ via IHC from plaque specimens of an additional 12 patients who underwent femoral (*n* = 5) or carotid (*n* = 7) endarterectomy. This yielded a total of 49 distinct patients who underwent femoral (*n* = 23) or carotid (*n* = 26) endarterectomy.

### Plaque processing into single-cell suspensions for scRNA-Seq or flow cytometry.

Immediately following excision in the operating room, femoral and carotid plaque samples were placed in saline and transported to the lab for processing into single-cell suspensions (ensuring time from plaque excision in the operating room to in-lab cell processing of < 30 minutes). We used a protocol previously validated for carotid endarterectomy specimens (19) that involved initial tissue processing on ice until the enzymatic digestion step. The protocol included washing the plaque thoroughly in DMEM (Corning, 10-013-CV) and digesting at 37°C for 1 hour in: 10 mL of DMEM with 10% FBS; type IV collagenase (MilliporeSigma, C5138) at 1 mg/mL final concentration; and DNase (MilliporeSigma, DN25), hyaluronidase (MilliporeSigma, H3506), collagenase type XI (MilliporeSigma, C7657) and collagenase type II (MilliporeSigma, C6885) each at 0.3 mg/mL final concentration. The mixture was filtered consecutively through 70 then 40 μm strainers, washed twice in PBS, and centrifuged at 300*g* for 8 minutes. Cell counts from the resulting single-cell suspensions were overall comparable for femoral versus carotid plaques, with femoral plaques exhibiting more variation in cell numbers isolated from individual plaques (Supplemental Figure 9). For scRNA-Seq analyses, immediately following generation of single-cell suspensions (the generation of which took < 2.5 hours after excision of plaques from operating rooms, given initiation of specimen processing < 30 minutes after plaque excision and < 2 hours required to generate single-cell suspensions), we then enriched suspensions for live cells and CD45^+^ cells. Dead cells were removed with a dead cell removal kit (Miltenyi Biotec, 130-090-101) according to manufacturer’s instructions. Suspensions were then enriched for immune cells with CD45^+^ selection using a CD45^+^ enrichment kit (Miltenyi Biotec, 130-045-801). These CD45^+^ enriched single-cell suspensions of plaque were then immediately transported on ice (within the same building) to load for sequencing reactions, ensuring < 3 hours’ time from plaque excision to sequencing reaction. For analyses of pooled samples (carotid samples 1 and 2, femoral samples 1–3), the same steps were performed at the same intervals, but CD45^+^ enriched specimens were cryopreserved immediately in liquid nitrogen and then thawed together and loaded for sequencing reactions within < 30 minutes of thawing. For flow cytometry analyses, CD45^+^ selection was not performed and suspensions were analyzed (or frozen on liquid nitrogen and then analyzed immediately after thaw) following the dead cell removal step.

### Sequencing.

For sequencing, the CD45^+^ enriched single-cell suspensions were converted to barcoded scRNA-Seq libraries using the Chromium Single Cell 3′ Library, Gel Bead, and Chip Kit from 10X Genomics. The Chromium Single Cell 3′ V3.1 Reagent (10X Genomics, PN-1000286) kit was used to prepare scRNA-Seq libraries. Reverse transcription, barcoding, complementary DNA amplification, and purification for library preparation were performed according to the manufacturer’s instructions. Sequencing was performed on a NovaSeq 6000 platform with Read 1 of 28 bp and Read 2 of 90 bp (Illumina). Sequencing reads were demultiplexed and aligned to the human GRCh38 transcriptome using the CellRanger V3 software (10X Genomics) (92). Filtering, unsupervised clustering, differential expression, and additional analysis were completed using Seurat V4 and ClusterProfiler packages for R (93–96).

### Quality control, filtering, integration, and clustering.

For analyses, femoral plaque sample matrices were imported into the Seurat v4 R package (93–96) and combined into a “femoral plaque” Seurat object. Likewise, carotid plaque sample matrices were imported and combined into a “carotid plaque” Seurat object. For both Seurat objects, cells were filtered for mitochondrial reads < 10%, 200 < nCount_RNA < 10,000, and 200 < nFeature_RNA < 10,000. Each Seurat object was then filtered to remove mitochondrial and ribosomal genes. Each Seurat object was also normalized and scaled and filtered to keep the top variable features (greatest standardized variance; *n* = 3,000) across the data sets. The objects were then merged using the Seurat merge command and integrated using the R package Harmony (97). The RunHarmony command was used to calculate harmonized dimension reduction components using the samples as the grouping variable, and doublet discrimination was performed. Principal components were then calculated, and an elbow plot was generated to select principal components to use for downstream analysis; here, 30 principal components explained most of the variation. UMAP dimensional reduction was then computed, followed by unsupervised clustering using the FindNeighbors and FindClusters Seurat functions, using the number of principal components mentioned above and a resolution of 0.3 (FindClusters), which captured distinct cell types empirically.

### Myeloid and lymphoid subclustering and batch correction.

Subclustering of myeloid cells was completed by first extracting the raw expression matrix from all myeloid cells for each sample using the GetAssayData function from the Seurat package. All carotid myeloid matrices were then combined into a carotid myeloid Seurat object, whereas all femoral myeloid matrices were combined into a femoral myeloid Seurat object. The carotid and femoral myeloid objects were then merged and, to correct for batch effects, integrated with Harmony (90) using sample as the grouping variable. UMAP dimensional reduction, downstream differentially expressed genes (DEG), and pathway analyses was then performed in the same manner as described above. This process was repeated with lymphoid cell data to create subclusters for lymphoid cells.

### Detection of DEGs.

Detection of DEGs between clusters was performed using the FindAllMarkers Seurat function, specifying return of significantly (Bonferroni *P*_adj_ < 0.05) upregulated genes with a log_2_ fold change (log_2_FC) threshold of 0.25. Cell types were assigned to clusters by evaluating gene expression of individual clusters using differential gene expression. For individual clusters, detection of DEGs between carotid and femoral plaque location was performed using the FindAllMarkers command specifying to return both positively and negatively changed genes and no log_2_FC or *P* value cutoffs. Genes with positive and negative log_2_FC values were used to identify upregulated genes in the carotid or femoral plaque location, respectively. For all DEG calculations, the RNA assay and data slot were used and performed using the default Wilcoxon rank-sum method.

### Pathway analyses.

Pathway analysis was completed using the ClusterProfiler R package (93–96). For comparison of enriched pathways between clusters, the compareCluster function was utilized on a matrix derived from the Seurat DEG analysis filtered for the top 100 positive, *P*_adj_ (< 0.05) genes that contained a column that indicated in which cluster each gene was upregulated. This analysis utilized the enrichGO database from ClusterProfiler (95) to return a table with enriched GO pathways in each cluster. For comparison of enriched pathways between carotid and femoral plaque locations within a specific cluster, the top 100 positive and negative genes from the Seurat DEG analysis by plaque location were separated to identify enrichment in either the carotid or femoral location, respectively.

### GSEA.

To identify subtypes of CD4^+^ T cells in our data set, we performed GSEA on clusters that were identified as CD4^+^ T cell clusters using DEG. GSEA was conducted using GSEA desktop software (98, 99), and our group’s previously curated gene sets of CD4^+^ T cell subtypes (100). Normalized enrichment scores were acquired using gene set permutations 1,000 times, and a cutoff *P* value of 0.05 was used to filter the significant enrichment results.

### Descriptive statistics.

Comparisons of cell clusters as a proportion of total CD45^+^ cells, myeloid cells, and lymphoid cells were performed using logistic regression; log ORs were determined using Fisher’s exact test and used to indicate comparatively higher or lower proportions in carotid versus femoral plaque. Based on 13 comparisons of proportions for overall clusters, 8 for myeloid clusters, and 9 for lymphoid clusters (a total of 30), we incorporated a conservative Bonferroni correction of *P* < 0.0017 (=0.05/30) to determine statistically significant differences in cell cluster proportions in carotid versus femoral plaque. For post hoc analyses, *P* < 0.05 was considered statistically significant.

### Flow cytometry of myeloid cells and T cells in plaque and blood of femoral and carotid endarterectomy patients.

To externally validate plaque site–specific findings related to macrophage, monocyte, and T cell phenotype observed in plaque from scRNA-Seq analyses, we prospectively enrolled 24 patients undergoing carotid (*n* = 15) or femoral (*n* = 9) endarterectomy. In these patients, we obtained blood preoperatively for processing into peripheral blood mononuclear cells (PBMCs) and flow cytometry of myeloid cells and T cells, and we surgically excised plaque, which was processed using the same procedures as for scRNA-Seq analyses (with the exception of sorting out live cells and nonleukocytes by flow cytometry rather than positive selection). For myeloid-focused flow cytometry of digested plaque specimens, cells were first gated on live, single cells, and macrophages were identified as CD11b^+^CD14^+^CD64^+^HLA-DR^hi^ cells before being further distinguished based on CCR2 expression. For PBMCs, monocytes were determined based on CD16 and CD14 expression, which was used to sort monocytes into classical, nonclassical, and intermediate populations. Subsequently, monocyte subpopulations were further distinguished based on CCR2 expression. T cell gating was performed on PBMCs and plaque first by identifying T cells as CD3^+^ and further distinguishing them by CD4 or CD8 expression. Of note, because T cells were the most common cell type in plaque in scRNA-Seq analyses, followed by macrophages and monocytes, we performed T cell phenotype–focused flow cytometry on samples from all 24 patients and additional myeloid-focused flow cytometry on a subcohort of patients with plaque with sufficient cell numbers (*n* = 11 who underwent carotid endarterectomy, *n* = 8 who underwent femoral endarterectomy; clinical characteristics in Table 2). To determine differences by plaque site, we performed pairwise 2-tailed *t* tests at an α of 0.05 comparing patients undergoing carotid versus femoral endarterectomy regarding: (a) proportions of macrophages in plaque that were CCR2^+^ for carotid versus femoral plaque, (b) blood monocyte CCR2 expression (as MFI), and (c) proportions of T cells in plaque and blood that were CD4^+^ and CD8^+^ (as a proportion of total T cells in that specimen). Flow cytometry reagents are included in Supplemental Table 1.

### Histological staging of plaque.

Plaques used for flow cytometry or scRNA-Seq analyses with sufficient tissue available following generation of single-cell suspensions were fixed in formalin and embedded in paraffin by the Pathology Core Facility at Northwestern University as above; they were then sectioned into 5 μm sections and mounted onto slides to undergo Masson’s trichrome staining for histological grading. Two trained pathologists performed the grading (shown in Supplemental Figure 10); they were blinded to plaque site and surgical indication, according to American Heart Association Classification (101) as done previously (19).

### In situ histologic exploration of leukocyte aggregates in femoral and carotid plaques.

For IHC analyses, postsurgical femoral and carotid plaque tissue from patients was prepared through the Pathology Core Facility at Northwestern University. Each tissue was fixed in formalin and embedded in paraffin, and 5 μm–thick slices were cut from each paraffin block and stained with H&E. Histology was reviewed by a trained pathologist blinded to plaque location. The pathologist screened whole H&E-stained slides cut from these formalin-fixed, paraffin embedded blocks of atheroma from 42 additional patients who underwent carotid or femoral endarterectomy, for the purpose of determining whether 5 or more cells with lymphoid appearance were present in any high-powered field; 12 of 42 samples (7 carotid, 5 femoral) met these criteria. Notably, none of the 42 samples contained adventitial tissue per the reviewing pathologist, consistent with endarterectomy technique of avoiding adventitial tissue (102). Contiguous slides from the same blocks of these 12 plaque samples subsequently underwent IHC. This included processing for 3, 3-diaminobenzidine–HRP (DAB-HRP) staining, counterstaining with H&E, and immunostaining for CD45, CD20, and CD3. Antigen retrieval and antibody staining was optimized at the Pathology Core Facility at Northwestern University. The stained sections were imaged utilizing 4× and 40× objectives on the bright-field mode via the Vectra 3 Automated Quantitative Pathology Imaging System (PerkinElmer) at the Immunotherapy Assessment Core at Northwestern University. The field of interest for each sample was chosen by a trained pathologist based on the available cellularity within each tissue. From there, the same cell cluster population within each sample was located under 40× for each staining available per sample (CD45, CD20, and CD3). In this 40× view, the DAB staining quantification was carried out in a blinded fashion by counting the number of cells of interest within this cell cluster population. We quantified cell cluster compositions individually to determine whether T cells and/or B cells were clustering in large aggregates.

### Statistics.

Differential gene expression analysis was conducted between identified clusters to find marker genes for each cell type using Wilcoxon rank-sum test. Bonferroni correction was used to adjust for multiple testing, and the cutoff *P* value of 0.05 after adjustment was set for all differential expression analysis. Pathway enrichment analysis was conducted with ClusterProfiler, which uses the Fisher’s exact test with a Bonferroni-corrected *P* value cutoff of 0.05. Comparisons of cell cluster proportions were performed using logistic regression; log ORs were determined using Fisher’s exact test to determine statistically significant (Bonferroni-corrected *P* value cutoff of 0.05) differences in cell type proportions in carotid versus femoral plaque. For flow cytometry, differences by plaque site were determined using 2-tailed pairwise *t* tests. *P* < 0.05 was considered statistically significant.

### Study approval.

Patient research, including written informed consent, was approved by the Northwestern University IRB (study nos. 205451 and 211811 used for this investigation).

### Data availability.

Data underlying this publication are available in Gene Expression Omnibus (GEO; accession no. GSE234077), and underlying code is deposited in GitHub with interactive links (https://github.com/Feinstein-Lab/single-cell-leukocyte-profiling-of-human-atheroma; branch name: main; commit ID: 840f3f8).

## Author contributions

JS designed the study, acquired data, analyzed data, and contributed writing and revising the manuscript. A Sinha designed the study, conducted experiments, acquired data, and wrote the manuscript. MD and SS acquired data, conducted experiments, and analyzed data. HA analyzed data and wrote the manuscript. IL analyzed data, drafted figures, and wrote the manuscript. KG, RN, CMW, RT, A Sunderraj, XW, and MS acquired data and conducted experiments. PD acquired data, analyzed data, and wrote the manuscript. SA, DMLJ, and SW analyzed data and wrote the manuscript. MV acquired data and analyzed data. R Saigusa, RG, JV, and R Sisk analyzed data. JL designed the study and analyzed data. KH, KPL, CG, and EBT designed the study, acquired data, and wrote the manuscript. MJF designed study, acquired data, analyzed data, and contributed to writing and revising the manuscript. JS was listed before A Sinha based on his primary role in bioinformatics analyses and writing.

## Supplementary Material

Supplemental data

Supporting data values

## Figures and Tables

**Table 1:**
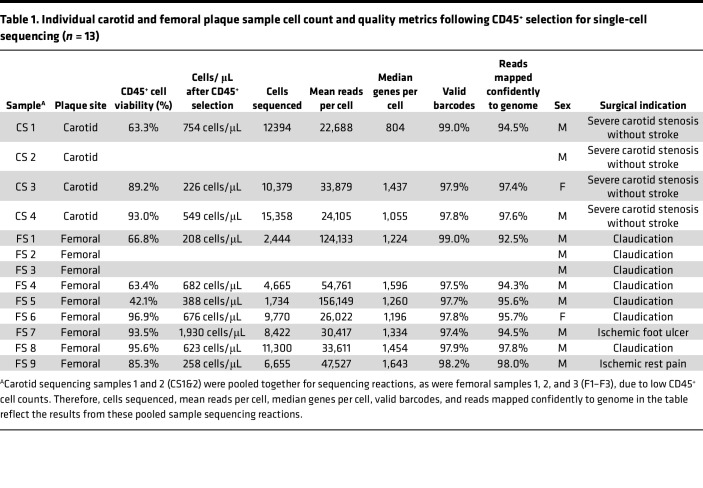
Individual carotid and femoral plaque sample cell count and quality metrics following CD45^+^ selection for single-cell sequencing (*n* = 13)

**Table 2:**
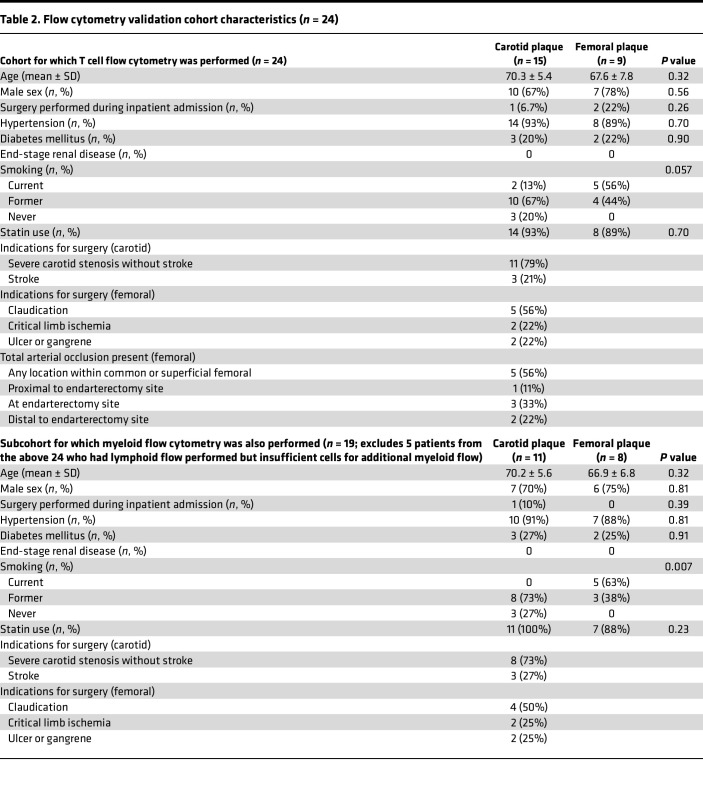
Flow cytometry validation cohort characteristics (*n* = 24)

## Version 1 07/20/2023

In-Press Preview

## Version 2 09/08/2023

Electronic publication
