# Supplementary material for: Single-cell profiling reveals inflammatory polarization of human carotid versus femoral plaque leukocytes
Source: JCI Insight. 2023 Sep 8;8(17):e171359. doi: 10.1172/jci.insight.171359 (PMC10544225; doi:10.1172/jci.insight.171359)
Supplement: Supplemental data [file jciinsight-8-171359-s231.pdf]

Supplemental Appendix

Supplemental Figures and Legends:

Supplemental Figure S1. Study population undergoing femoral (N=23) or carotid (N=26) endarterectomy.

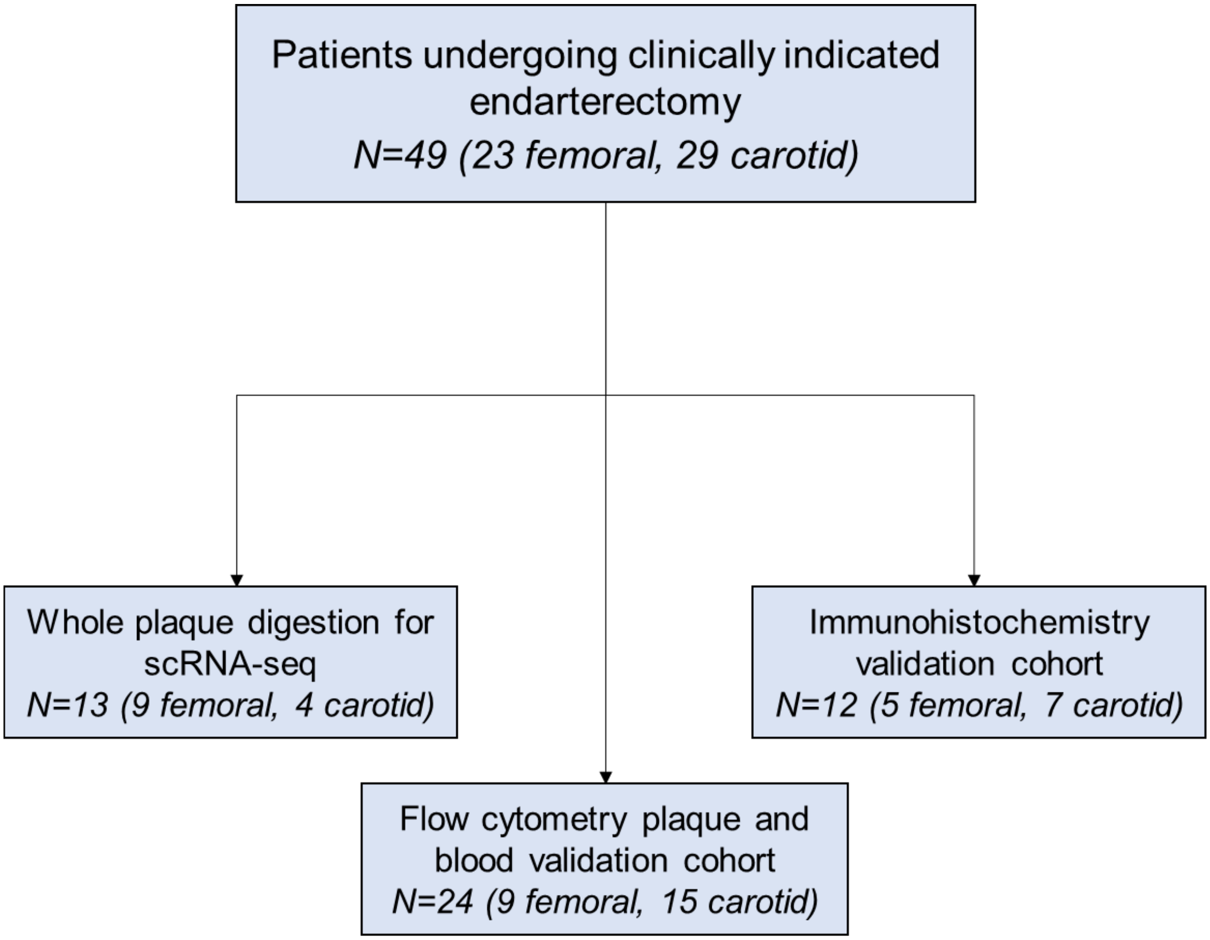

Supplemental Figure S2. Femoral and carotid plaque cell sequencing-related quality metrics and filtration of cells for analyses

Legend. Carotid and femoral cells were analyzed and filtered based on <10% mitochondrial RNA, 200-2500 genes per cell, and 200-10000 unique molecular identifiers (UMIs) per cell.

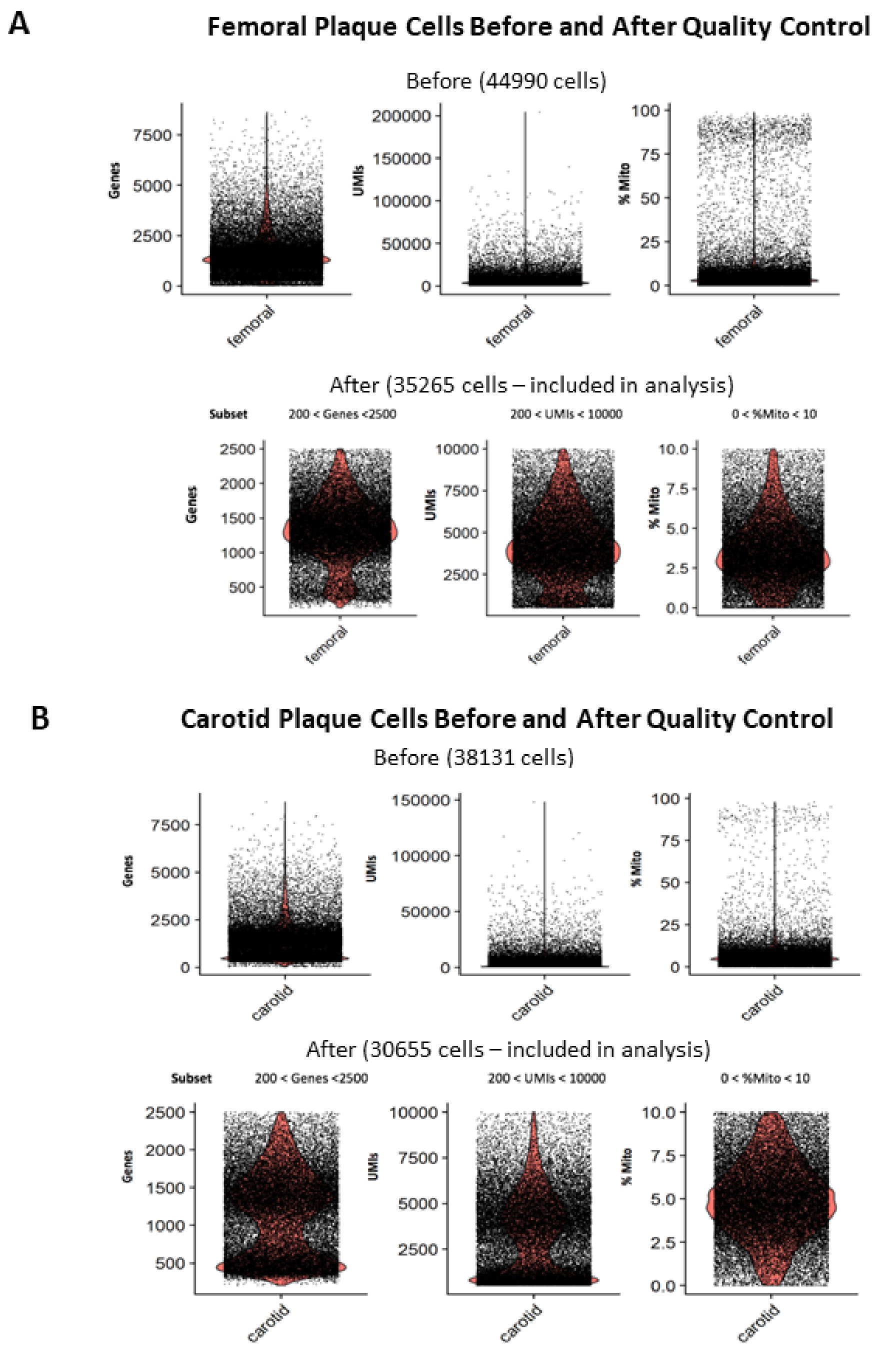

Supplemental Figure S3. CCA Clustering and UMAP visualization of CD45 positive-selected cells from femoral and carotid plaque, visualized separately by individual sample. ***Legend:*** CCA Clustering and UMAP visualization of all femoral (N=9) and carotid (N=4) plaque samples for which scRNA-seq was performed, with the overall clustering depicted **(A)** along with **(B)** separate plots for each sample. Carotid samples 1&2 were pooled together, as were femoral samples 1, 2, & 3, due to low CD45+ cell numbers; indications for surgery were the same (asymptomatic carotid stenosis for C1&2, chronic claudication for F1-3) among pooled cells. **(C)** Comparative boxplots (with individual sample points) representing cluster proportions for carotid and femoral samples.

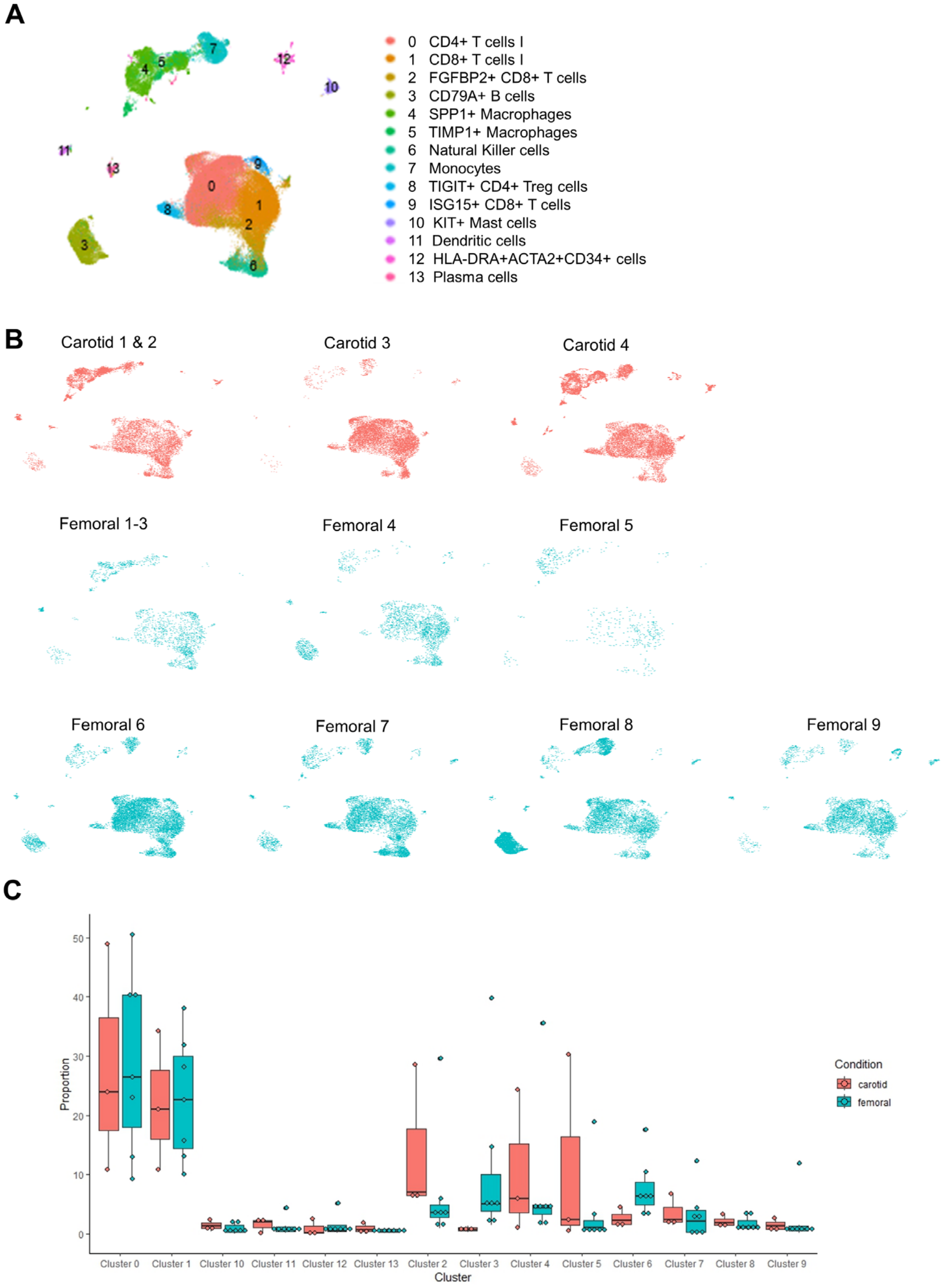

Supplemental Figure S4. CD45 positive-selected CCA Clusters of femoral (n=9; 35265 cells) and carotid (n=4; 30655 cells) atherosclerotic plaque show distinct gene expressions. (A) Heatmap colored based on log_2-fold expression levels. (B) Feature plot of top marker genes per cluster.

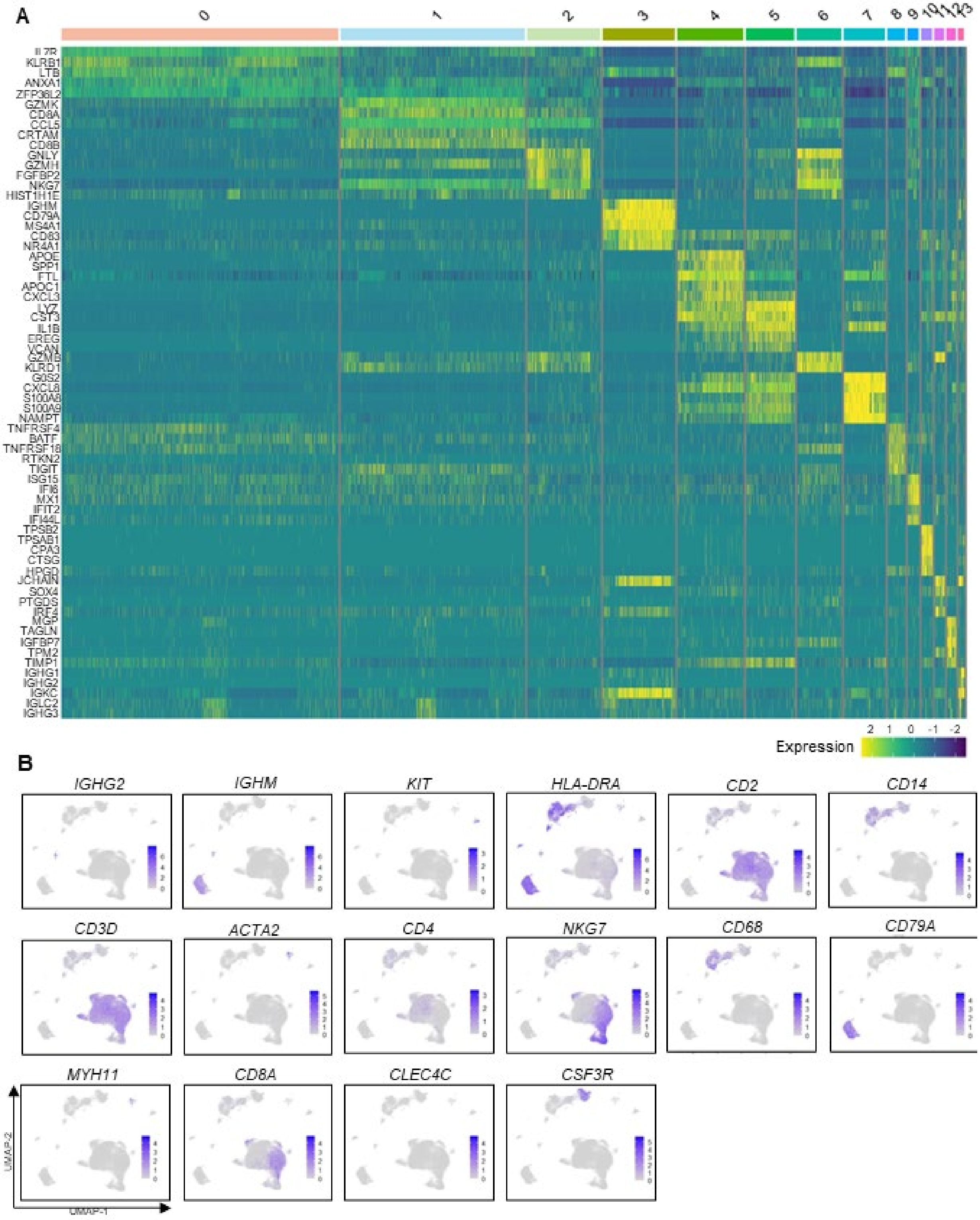

Supplemental Figure S5. Automatic cell annotation via CellTypist on femoral (n=9; 35265 cells) and carotid (n=4; 30655 cells) atherosclerotic plaque of CD45 positive-selected cells.
Legend: (A) UMAP visualization of the entire samples colored by CellTypist annotations, and (B) separated between carotid and femoral samples. (C) Corresponding table of comparing cell proportions in carotid vs. femoral plaque based on CellTypist annotations.

A

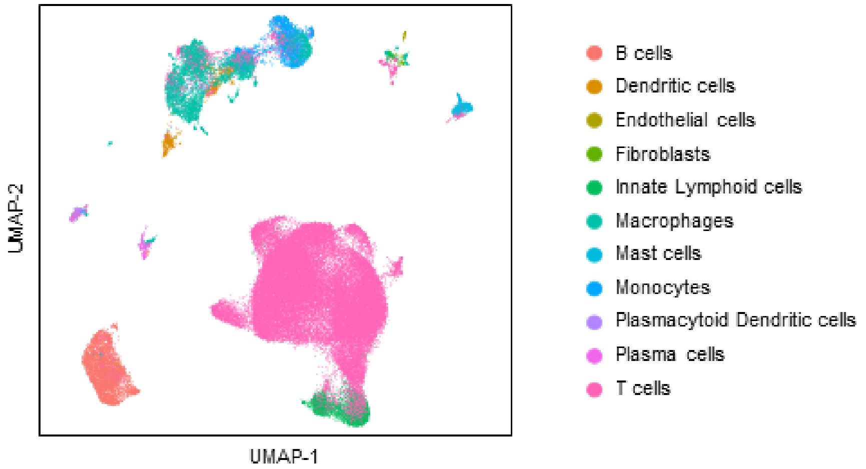

B

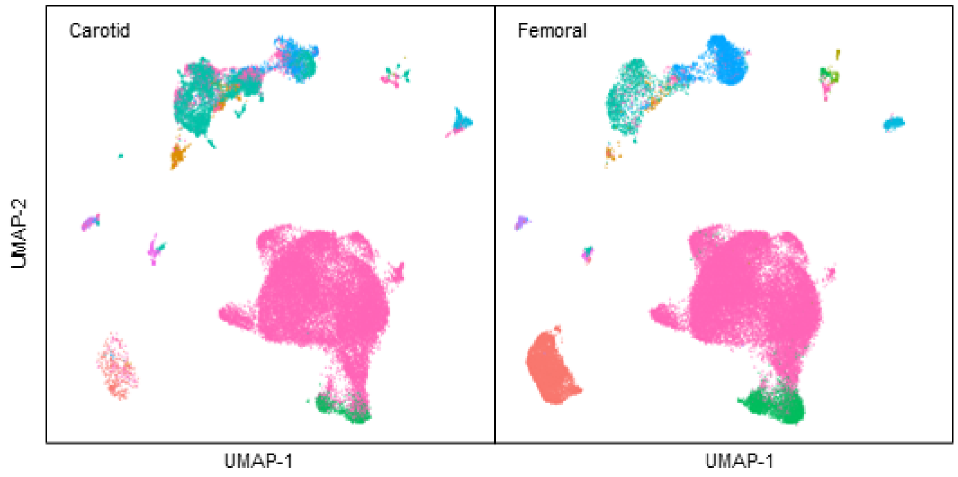

C

	Carotid N (n=30655), % carotid cells (estimate ± SE)	Femoral N (n=35265), % femoral cells (estimate ± SE)
B cells	252 (0.8 ± 0.05)	5221 (14.8 ± 0.2)
Dendritic cells	849 (2.8 ± 0.05)	207 (0.6 ± 0.04)
Endothelial cells	2 (0.01 ± 0.005)	76(0.2 ± 0.02)
Fibroblasts	2 (0.01 ± 0.005)	89 (0.3 ± 0.03)
Innate Lymphoid cells	651 (2.1 ± 0.08)	2321 (6.6 ± 0.1)
Macrophages	4205 (13.7 ± 0.2)	1355(3.8 ± 0.1)
Mast Cells	304 (1.0 ± 0.06)	379 (1.1 ± 0.05)
Monocytes	1253 (4.1 ± 0.1)	2027 (5.7 ± 0.1)
Plasmacytoid Dendritic cells	292 (1.0 ± 0.06)	237 (0.7 ± 0.04)
Plasma cells	211 (0.7 ± 0.05)	91 (0.3 ± 0.03)
T cells	22634 (73.8 ± 0.3)	23262 (66.0 ± 0.3)

Supplemental Figure S6. Myeloid subset of scRNA-seq data for femoral (n=9; 8941 cells) and carotid (n=4; 4461 cells) atherosclerotic plaque shows distinct gene expressions. (A) Heatmap colored based on log2-fold expression levels. (B) Feature plot of top marker genes per cluster.

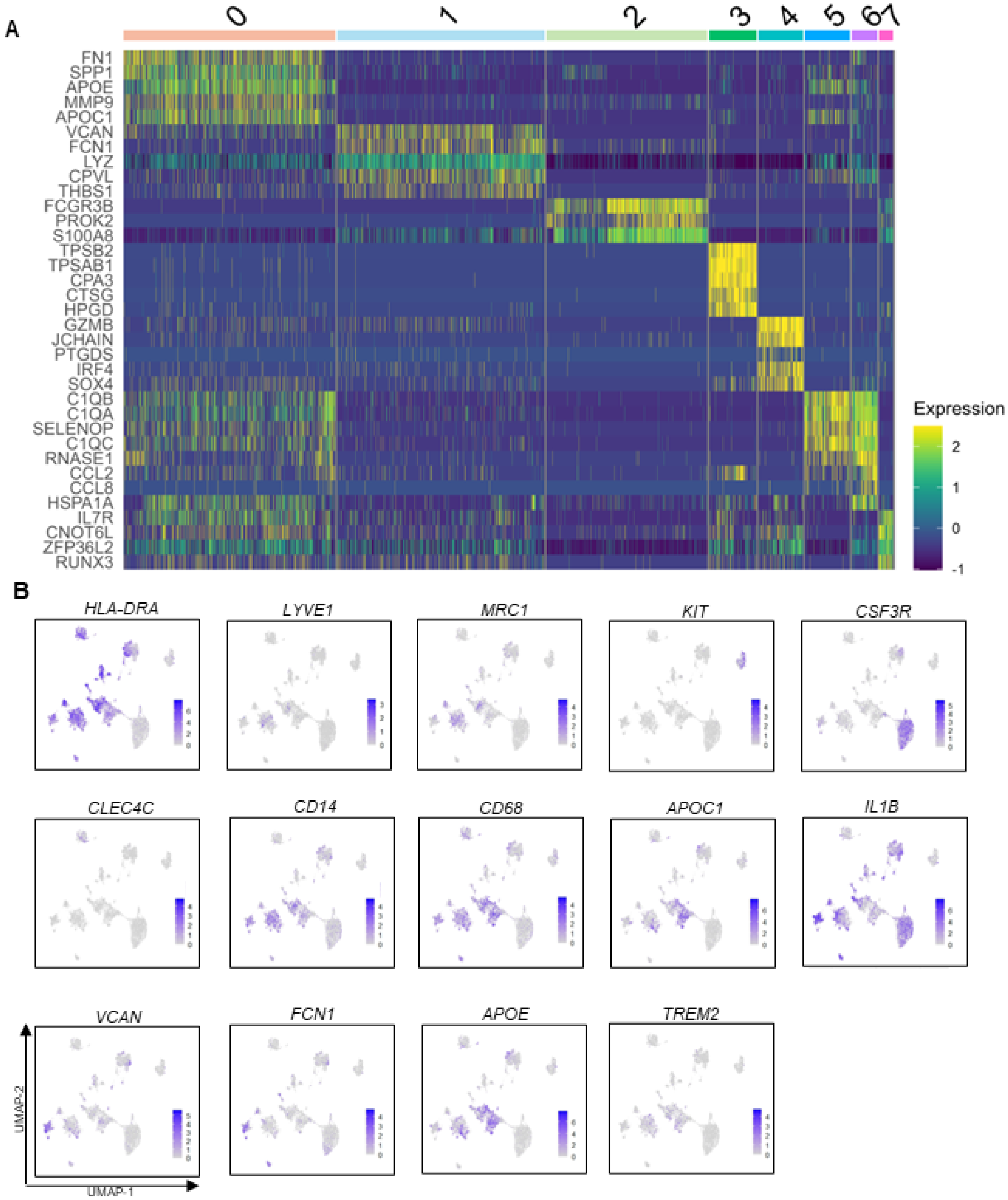

Supplemental Figure S7. Lymphoid subset of scRNA-seq data for femoral (n=9; 21386 cells) and carotid (n=4; 30430 cells) atherosclerotic plaque shows distinct gene expressions. (A) Heatmap colored based on log2-fold expression levels. (B) Feature plot of top marker genes per cluster. (C) Volcano plot of highly expressed genes in the lymphoid subset of carotid and femoral plaque samples.

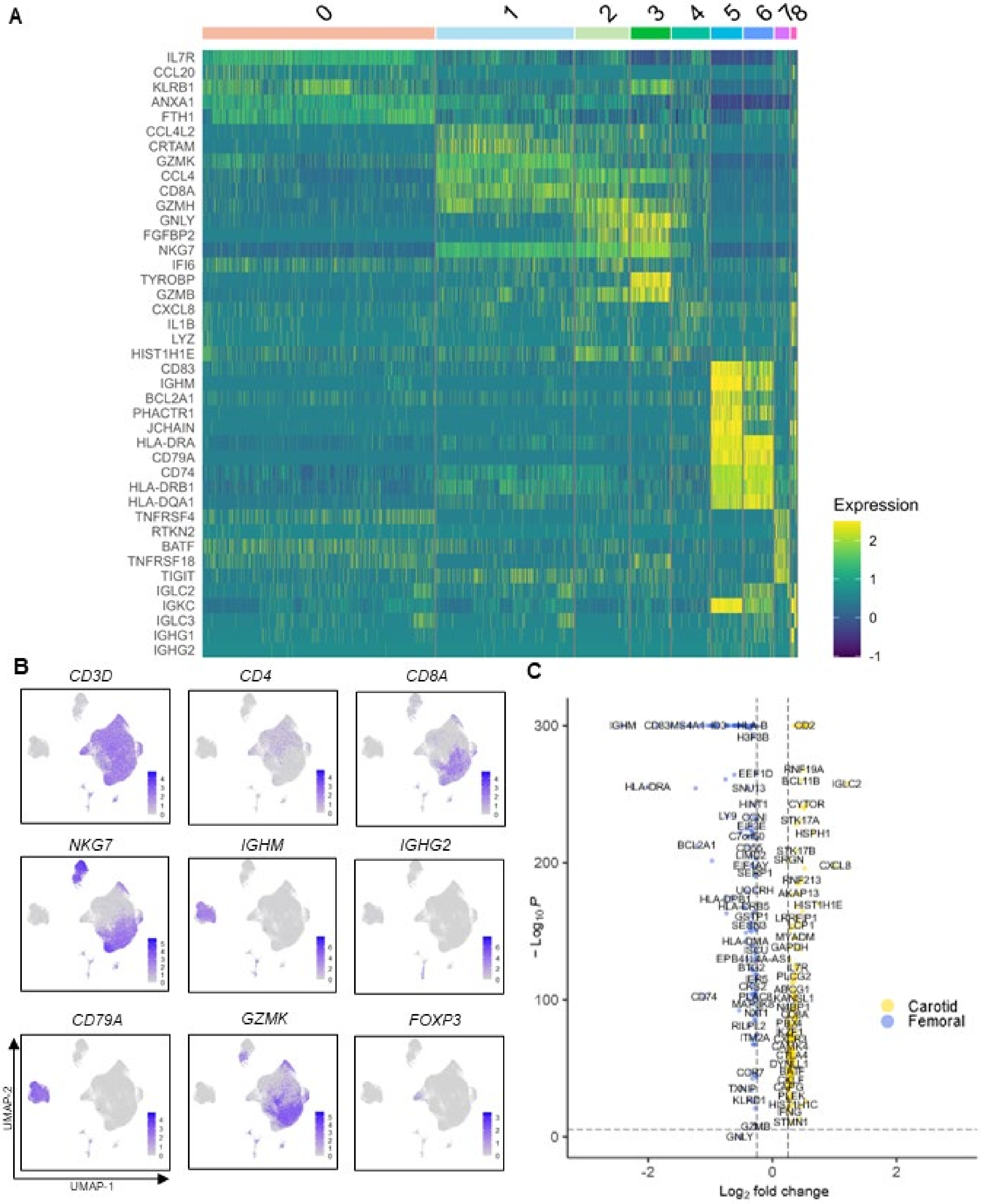

Supplemental Figure S8. Flow cytometry gating strategy for T cells.
Legend. Cells were first gated on live, single cells. T cells were identified by CD3 expression further distinguished based on CD4 and CD8 expression. Representative flow plots of plaque CD4+ and CD8+ T cells from carotid or femoral endarterectomy patients.

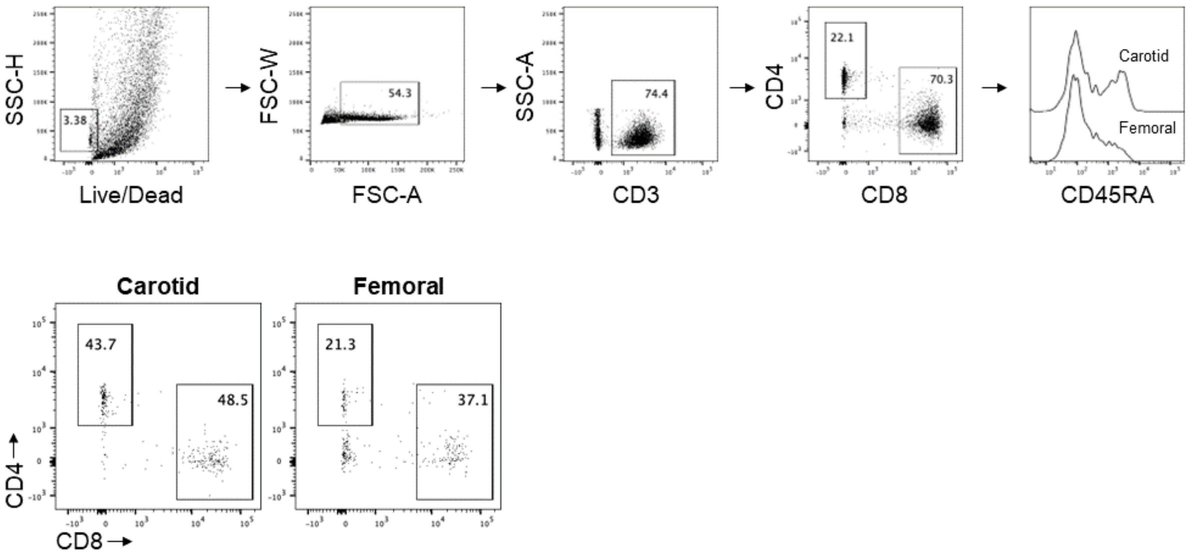

Supplemental Figure S9. Cells and cells/mg in single-cell suspensions of plaque by site and clinical acuity prior to CD45 selection for scRNA-seq or prior to undergoing flow cytometry

Legend. Single-cell suspensions of whole femoral and carotid plaques digested into single-cell suspensions for flow cytometry or scRNA-seq had estimated overall cell numbers, estimated based on visualization under a hemocytometer prior to CD45 positive selection, that did not differ significantly (Panel A), although the range of cells per plaque was larger among femoral plaques. Plaque cells (Panel B) and cells/mg tissue (Panel C) likewise did not differ by acuity of clinical indication for surgery. 27 out of 37 plaques digested into single-cell suspensions had estimated cell counts prior to CD45 selection recorded and were thus included in the analysis. Fishers exact test with a p=0.05 to determine significance was performed for all pairwise comparisons; NS indicates not significant. Acute PAD was defined as acute limb ischemia or other severe/acute ischemic complications including foot ulcer or ischemic rest pain.

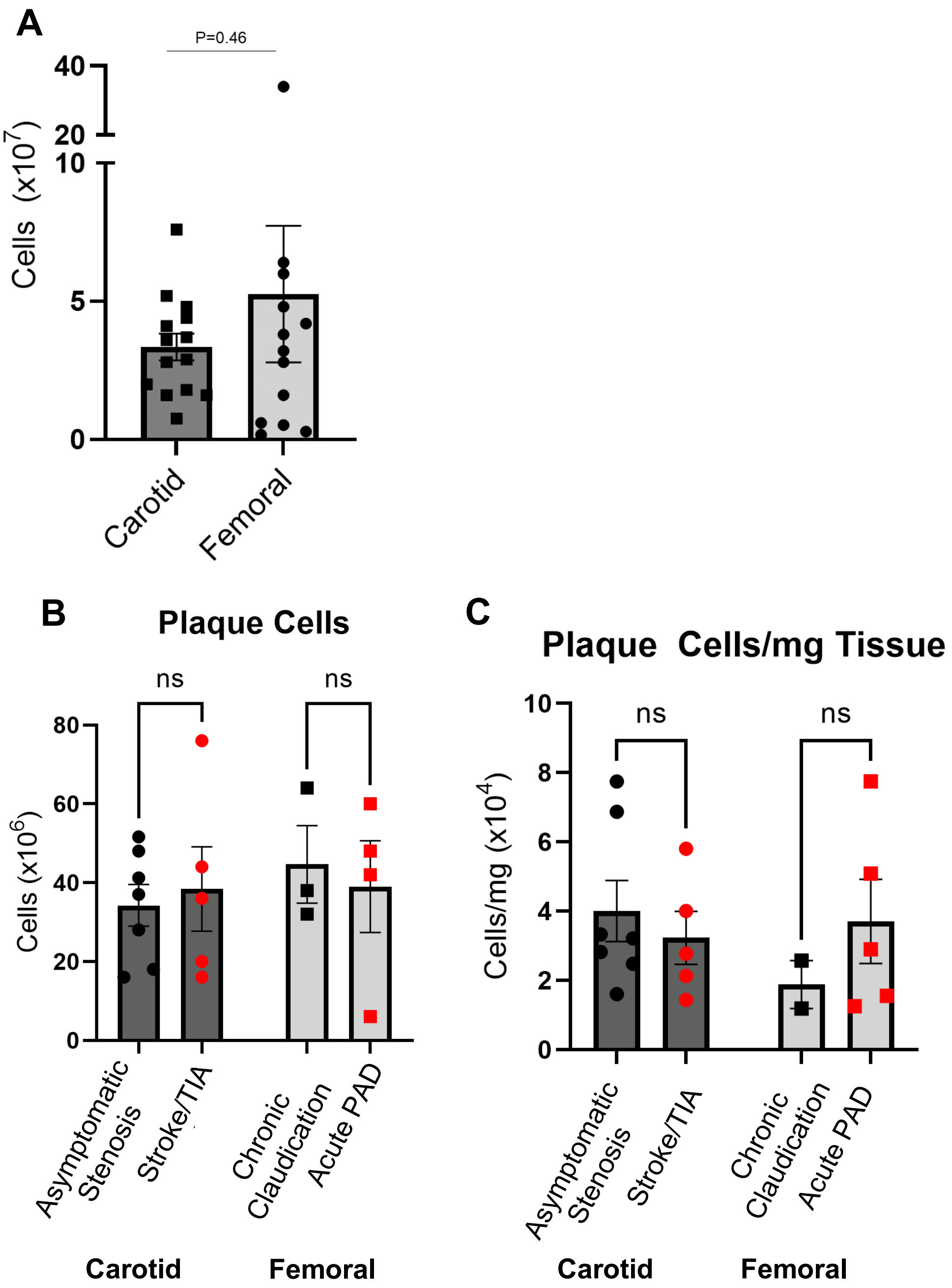

Supplemental Figure S10. Carotid and Femoral plaque histologic grading

Legend. Two trained pathologists graded carotid and femoral plaques used for flow cytometry or scRNA-seq according to American Heart Association classifications, blinded to plaque site and surgical indications. All plaques analyzed were at histologic stage V or VI, with the majority of femoral (80%) and carotid (73%) plaques being stage VI and the remainder stage V. Because whole plaques were digested to optimize cellular yield of single-cell suspensions for scRNA-seq and flow cytometry analyses, 21 out of 37 plaques had sufficient tissue for histologic analyses after single-cell suspensions were generated; these plaques were subsequently analyzed by the two pathologists after undergoing Masson's Trichrome Staining.

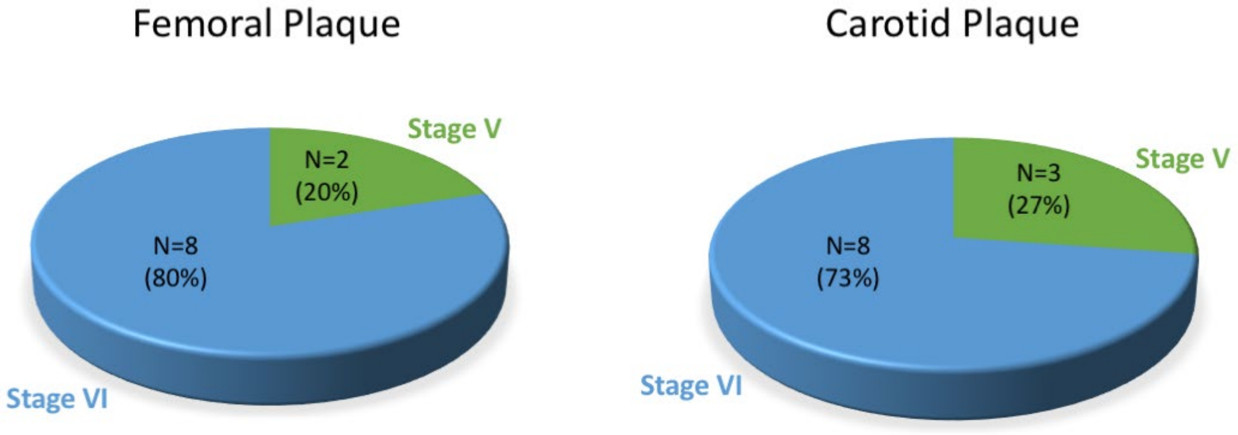

Supplemental Tables

Supplementary Table T1. Flow cytometry reagents and resources

REAGENT or RESOURCE	SOURCE	IDENTIFIER
Antibodies		
AF700 anti-human Siglec6	R&D Systems	Cat #: FAB2859N
APC anti-human AXL	R&D Systems	Cat #: FAB154A
APC/Cy7 anti-human HLA-DR	BioLegend	Cat #: 307618
BV421 anti-mouse/human CD11b	BioLegend	Cat #: 101251
BV605 anti-human CD141	BioLegend	Cat #: 344117
BV650 anti-human CD11c	BioLegend	Cat #: 301637
BV711 anti-human CX3CR1	BioLegend	Cat #: 341629
FITC anti-human CD64	BioLegend	Cat #: 305005
PE anti-human CD192 (CCR2)	BioLegend	Cat #: 357205
Pe/Cy7 anti-human CD14	BioLegend	Cat #: 301813
PE/Dazzle 594 anti-human CD56 (NCAM)	BioLegend	Cat #: 362543
PerCP/Cy5.5 anti-human CD16	BioLegend	Cat #: 302027
TruStain FcX (Human Fc Receptor Blocking Solution)	BioLegend	Cat #: 422302
Zombie Aqua Fixable Viability Kit	BioLegend	Cat #: 423101
